# We can be heroes: MLA’s leadership journey(s)*

**DOI:** 10.5195/jmla.2017.127

**Published:** 2017-01

**Authors:** Mary Joan (M.J.) Tooey

## Abstract

**Objective:**

Are there key attributes of leaders? Extrovert versus introvert? Charismatic? Detail oriented? Visionary? How do past leaders of the Medical Library Association (MLA) stack up? What leadership skills will MLA’s leaders need in a complex information future? Leadership attributes of MLA’s past and current presidents were studied to determine the common characteristics shared among these leaders. An examination of the leadership literature identified critical leadership characteristics essential to successful future leaders. MLA’s past, current, and future leadership development efforts were examined. Finally, all members were encouraged to consider leadership with a small “l” and become leaders based on their own strengths, interests, and environments.

**Methods:**

A text analysis was performed on past presidential profiles, the past twenty-five years of MLA presidents were surveyed, and conversations with MLA’s current presidents were held to determine commonalities among leadership characteristics. These were compared and contrasted with characteristics in the current leadership literature regarding the qualities of future leaders.

**Results:**

The text analysis of past presidential profiles was not particularly revelatory regarding leadership qualities of early MLA presidents although several generalized traits emerged including collaborative traits; management traits such as effectiveness and efficiency, innovation, and vision; personal traits such as humor and energy; and finally, a passion for the work were revealed. These aligned with traits identified in the survey of the past twenty-five years of MLA presidents and with the thoughts of the president-elect, president, and past president. Additional qualities identified were communication skills, political acumen, creativity, courage, and respect for the opinions and concerns of all members. MLA’s current leadership programs were reviewed in the context of examining traits needed by leaders of the future. A lack of focus on the needs of middle managers and the development of individual leadership skills was identified.

**Conclusions:**

As an organization, MLA should focus on leadership development in contrast to management training to prepare members as leaders in careers and work that may be vastly different than current situations. Equipping members with the skills enabling them to lead and thrive in these diverse situations, whether as the heads of programs or middle managers, or exploring and empowering individual leadership development while maintaining a passion for the profession, will be essential.

## SUPPLEMENTAL FILES

Appendix A Transcript of the 2016 Janet Doe Lecture

Appendix B PowerPoint slides from the 2016 Janet Doe Lecture

