# Supplementary material for: We can be heroes: MLA’s leadership journey(s)
Source: J Med Libr Assoc. 2017 Jan;105(1):88. doi: 10.5195/jmla.2017.127 (PMC5234446; doi:10.5195/jmla.2017.127)
Supplement: Appendix A [file jmla_jan17_tooey_appa.pdf]

JMLA Journal of the Medical Library Association

Supplemental content to
J Med Libr Assoc. Jan;105(1): dx.doi.org/10.5195/jmla.2017.127
www.mlanet.org
© Tooey 2017

We can be heroes: MLA's leadership journey(s)

Mary Joan (M.J.) Tooey, AHIP, FMLA

APPENDIX A

Transcript of the 2016 Janet Doe Lecture

SLIDE 1: TITLE

While Barbara A. Epstein, AHIP, FMLA, exits I will say, obviously my contract didn't hold because there were supposed to be Mimosas and Bloody Marys on the tables to kind of warm up this audience, but I will talk to them after it's done. I don't get a redo so it's kinda, "Oh, man, this is bad." And all of you didn't take my advice to sleep in this morning, so I am a little bit concerned about that.

Good morning. Bonjour.

What a pleasure it is to have Barbara Epstein introducing me today. I have known and been friends with Barbara for decades. Literally decades. One of the facts about the Doe is that the previous Doe lecturer introduces you. And throughout the year, any time I would talk to Barbara, she would say "be nice to me because I have to introduce you." So, I must have done a good job since that was a lovely introduction.

SLIDE 2: BARBARA AND MJ

So our first trip into the "Wayback Machine"…When I was in library school at the University of Pittsburgh taking the health sciences bibliography course (yes, that is what it was called), Barbara was a relatively new librarian at Western Psychiatric Institute and Clinic, better known as Western Psych. Many assignments took me there. At that time, she was the young librarian we all aspired to be. Now, she is the

Supplemental content to
J Med Libr Assoc. Jan;105(1): dx.doi.org/10.5195/jmla.2017.127
www.mlanet.org
© Tooey 2017

experienced library leader I still admire. She has been so gracious, encouraging, and supportive over this past year, just as she was when I first met her thirty-five years ago. Thank you again, Barbara.

SLIDE 3: THANK YOUS

All too often, we wait until the end of a presentation to thank those who have been instrumental and supportive. Through readings, example, and feedback, these people have supported me with their generosity, intelligence, and insights regarding leadership; with their support for this lecture; and throughout my entire career. And in particular, the last two people have supported me with a tremendous amount of love. One of them is in the audience today. So, on the screen you are seeing a rolling list of all those who helped me in some way on my Janet Doe journey. It takes a village to deliver a Janet Doe Lecture, and I thank you all.

SLIDE 4

The Medical Library Association (MLA) has made me cry twice. The first time was in 1997, when I received the Estelle Brodman Award for Academic Medical Librarian of the Year, and those were tears of joy and excitement. The second time I cried was when I learned I was to be the 2016 Janet Doe lecturer. The Janet Doe lecturer for any given year is named more than a year in advance. Why you ask? This is in order to allow sufficient time to prepare the lecture and to build the level of panic as the date approaches. So those indeed were tears of pure terror.

The Janet Doe Lecture has been delivered by luminaries and leaders in our field who have provided eloquent and elegant insights into our profession. They have served as inspirations and guideposts for our careers. To look back on the list of lecturers and their lectures is a humbling experience. And to know one’s name is going to be added to that list may actually be the real reason I cried when I learned of the honor. And an honor it is.

JMLA Journal of the Medical Library Association

SC

Supplemental content to
J Med Libr Assoc. Jan;105(1): dx.doi.org/10.5195/jmla.2017.127
www.mlanet.org
© Tooey 2017

SLIDE 5

So, I think Nina W. Matheson, AHIP, FMLA, may have said it best in her 1994 Doe Lecture when she said, "Every Doe lecturer since, has disclaimed professional qualifications for writing history or philosophy…All have written about what they hold nearest and dearest to their professional hearts, seeking to inform, to provide insight, to inspire, and even to entertain" [1].

So, if you will indulge me, I would like to share some insights regarding my Doe journey.

SLIDE 6

One of my inner medical librarianship demons is that I have always envied colleagues who have deep expertise in specific areas like teaching and learning, outreach, expert searching, systematic reviews, or metadata management, anything like that—the people who go deep into their expertise. I, however, am a sampler, preferring to know a little bit about a lot and looking at things broadly. This perspective supports examining interrelationships, connections, and trends across wide areas of interest, looking for commonalities and relationships. It would probably make me great *Jeopardy* contestant. A colleague once introduced me as one of the most "curious" people she knows. I chose to interpret that as meaning inquisitive…others might interpret it as odd. On reflection, this learning style suited my approach to this lecture.

SLIDE 7: CONFESSIONS

And now for some truths and confessions. Before we start our Doe journey, I have some things to say.

1. I never met Janet Doe or any other early MLA luminary founder, and although I have known many wonderful contemporary leaders, I never met any of those more experienced older people. I did once receive a nice note from Estelle Brodman. And I believe I have had a supernatural encounter with Marcia C. Noyes. More on that later.

Supplemental content to
J Med Libr Assoc. Jan;105(1): dx.doi.org/10.5195/jmla.2017.127
www.mlanet.org
© Tooey 2017

2. I did not read all the previous Doe Lectures. That had been alluded to in previous lectures as a tradition. Sorry. I hope this won't cause you to rush the stage and throw me out. However, I did read some pertinent ones.
3. And the final truth is that when you start preparing the Doe Lecture, you have grand ideas regarding how you are going to present the lecture and wow the audience. As it gets closer and closer to the lecture, those frills start to fall away. So in what I am sure will be another disappointment, there will be no shadow puppets, there will be no costume changes, there will be no holograms, and worst of all, no interpretive dance as I originally planned.

SLIDE 8

There has been an interesting personal synchronicity to this year's lecture. This is the fiftieth anniversary of the establishment of the Janet Doe Lecture. The first lecture was actually given in 1967 by Gertrude Annan, another luminary I never knew. This is my thirtieth MLA meeting. It has been ten years since I had the honor of serving as MLA's president in what is fondly referred to as the "Tooey Reign of Terror." And, on the day I began my six-week administrative assignment to do research for this lecture thanks to the generosity of the leadership at the University of Maryland, Baltimore, David Bowie, one of my favorite artists and pop icons, died, which led me today to our title, "We Can Be Heroes (and those of you who know David Bowie know that his 'Heroes' song is one of my favorites): MLA's Leadership Journey(s)." So MLA's heroes, our presidential leaders and our professional and personal leadership journeys, are what we are going to examine today.

SLIDE 9: REPEAT TITLE

Let us begin our journey.

JMLA Journal of the Medical Library Association

Supplemental content to
J Med Libr Assoc. Jan;105(1): dx.doi.org/10.5195/jmla.2017.127
www.mlanet.org
© Tooey 2017

Leadership and the qualities of leaders are of interest to me, as they have been to every culture, every society, every institution, every organization, and most people. Joshua Rothman wrote in his February 29, 2016, *New Yorker* Critic at Large column, entitled "Shut Up and Sit Down: Why the Leadership Industry Rules," luminaries as diverse as Plato, Confucius, and Machiavelli have thought about leadership and the qualities of leadership [2]. He cites a Kinsey report stating that American companies spend almost 14 billion, billion with a "b," dollars annually on leadership training. The age-old questions of whether a leader is born or made, whether there are shared characteristics among leaders, and the qualities of great leaders tease our minds. Broadly researching leadership and leadership characteristics, and then examining these within the context of our own professional home, career, and personal development offered great opportunities. Based on readings and research, I found this is also a topic of great interest to many in our library communities as our association and profession strive to remain relevant and heard in the fast-paced information world in which we work and live. And it is not just about remaining relevant and heard, but leading as well. How do we grow and nurture new leaders? Are there things we can learn from our association past? 2016 seemed a great year to reflect on this, with the cacophony of the US elections all around us, bludgeoning us with evidence of lack of leadership.

Respecting the intent of this lecture with its focus on history and perspective on our profession, the first step of our MLA leadership journey today includes analysis of leadership traits of our past leader-heroes. Additionally, surveying the past twenty-five years of MLA presidents and interviewing current MLA leaders and MLA executive directors helped to identify historical and evolutionary commonalities in our association's perception of leadership. The next leg in the lecture journey takes a brief look at how MLA has, is, and will be supporting leadership development. A brief review of the characteristics experts say will be necessary for our personal and professional survival and futures will be next, and finally, we'll take some time to reflect on our own ability to lead. By the time we reach the end

Supplemental content to
J Med Libr Assoc. Jan;105(1): dx.doi.org/10.5195/jmla.2017.127
www.mlanet.org
© Tooey 2017

of this Doe journey, we will know we ALL can be heroes. We will be creating a mosaic of leadership journeys.

In a frequently used quotation by Lao Tzu, who was actually the father of servant leadership, the concept of servant leadership, he said, "The journey of a thousand miles begins with one step" [3]. Please take a step and join me on these journeys this morning.

SLIDE 10: KOTTER

A seminal article by Harvard Business School Professor John Kotter, entitled "What Leaders Really Do," published in the *Harvard Business Review* in 1990 [4], proposed leadership and management are different but complementary systems of action, as can be seen on this slide. In a changing world, they must coexist. His major point was managers cope with complexity, while leaders cope with change.

Management	Leadership
Planning/budgeting	Vision/strategies
Organizing/staffing	Aligning
Controlling	Motivating

Not all good managers are good leaders, and not all leaders are good managers—that is appropriate and makes sense. The great Warren Bennis, who studied corporate leadership said, "Managers are people who do things right and leaders are people who do the right thing" [5].

While the buzzword of this lecture is leadership, we must acknowledge the relationship between leadership and management, and accept that some management traits will slip in and that will be okay. After all, we are talking about leading an association, our own organizations, and our personal leadership journeys, where a strong balance between management and leadership is needed.

JMLA Journal of the Medical Library Association

Supplemental content to
J Med Libr Assoc. Jan;105(1): dx.doi.org/10.5195/jmla.2017.127
www.mlanet.org
© Tooey 2017

SLIDE 11

The title of the 2008 Janet Doe Lecture by Thomas Basler, FMLA, was "There Are No More Giants: Changing Leadership for Changing Times" [6], and one would be led to believe by that title he was saying there are no more leaders in our profession. Knowing Tom, and because he is probably psychic, this title was intentionally meant to provoke the 2016 Janet Doe lecturer. His lecture is not available in the *Journal of the Medical Library Association (JMLA),* but he kindly shared his slides with me. He did explore some of our more illustrious MLA forbears and the amazing impact they had on the shaping of our profession, but his lecture moved into a comprehensive review of the foundation and growth of our profession, and the impact of changes in our environment, thus saving himself from my future wrath. He told us the things we individually need to do to survive and thrive in these changing environments. He told us we are in control of our destinies…Perhaps we shouldn't wait for "giants" to bail us out. Many of the terms and attributes he used we will hear over and over again this morning.

However, he did not explore or contemplate the "why" behind MLA leadership. So, why were our early leaders, our leaders? How to look back at the past? How to discover the power and characteristics of our past leaders? We can't talk to them or feel their personal magnetism, although a few of them have oral histories, so we can hear their voices. And though it is tempting to develop a revisionist viewpoint of their greatness through our modern lens, they weren't all wonderful nor would we even recognize some of their names.

SLIDE 12

As Rachael K. Anderson, AHIP, FMLA, observed in her landmark 1989 Doe Lecture, "Reinventing the Medical Librarian" [7], which is as fresh and relevant today as it was back then, our association forbears were confronted by gender bias and racism—and reacted not always in a way of which we would be proud. The two-part series of *JMLA* articles, "Race and Librarianship," by Carolyn Lipscomb, AHIP,

Supplemental content to
J Med Libr Assoc. Jan;105(1): dx.doi.org/10.5195/jmla.2017.127
www.mlanet.org
© Tooey 2017

FMLA, published in 2004 and 2005, are a must read [8, 9]. We expect our leaders to be embodiments of our better selves, and that may not always be the case.

SLIDE 13

In the mid-20th century, MLA was a smaller professional organization. In 1940, there were 291 individual members and 147 institutional ones. These early leaders and heroes were great because they were establishing a profession and an association we nurture today. And they knew of or knew each other: they knew strengths, they knew weaknesses, and they knew accomplishments. Their main form of communication was writing letters. The prevalent information technology was the typewriter. However, if nothing else they had the skills, the vision, and as J. Michael Homan, AHIP, FMLA, put it in his presidential inaugural address, "A Passion for the Profession" [10]: They were doers. They assumed the mantle of responsibility. And they adroitly combined leadership and management qualities.

Did they exhibit leadership traits we might recognize from the Kotter article or today's leadership literature? And how to find out?

Before we climb into the MLA Leadership "Wayback Machine" and analyze our past leaders, I would like to share two side trips I took on my leadership exploration journey into our foundational past. As I mentioned, I enjoy scanning broadly and bringing disparate pieces together, and every now and then I would take a side trip. My side trips were as a result of wondering just how to find out more about our presidential leaders. One story involves a trip and the other, a ghost.

SLIDE 14

The trip occurred last November, when the MLA recognition plaque was unveiled on Chestnut Street in Philadelphia. It recognizes the founding of MLA in 1898 by physicians George M. Gould and Sir William Osler AND Margaret R. Charlton, LIBRARIAN. Although there were four physicians and four librarians

Supplemental content to
J Med Libr Assoc. Jan;105(1): dx.doi.org/10.5195/jmla.2017.127
www.mlanet.org
© Tooey 2017

in attendance, these three were the primary movers and shakers. Seeing the use of the word "librarian" was, I have to say, an emotional moment. It was recognition that we were involved with the founding of a profession, our profession, and that it wasn't just founded by a group of male physicians wanting to share medical knowledge. Margaret Charlton, who was a Canadian, was an equal partner. We will never know specifically what was said, but we do know they were leaders and visionaries.

Everyone should read at least one history of MLA to learn more about the details of our founding. In particular, Frances Groen's 1998 article, written for our centennial, "Three Who Made an Association," is an excellent focus on our three founders [11].

MLA remained a physician-led association until 1933.

Does anyone know what happened then? One of the four founding librarians in Philadelphia, Marcia Crocker Noyes was elected the first female, nonphysician president of MLA [12, 13]. And that is why our highest honor; the Marcia C. Noyes Award is named for her. While the dissemination of medical knowledge was one of the main reasons MLA was founded, Marcia Noyes made it happen.

And in kind of in a parallel incident, isn't it interesting that the first nonphysician female leader of the National Library of Medicine was named last week? We should also probably mention Carla Hayden as the first Librarian of Congress—female, woman of color. So cool.

Marcia C. Noyes was a remarkable, unobtrusive, and humble woman, and not only was she one of the founding librarians, but she also edited the *Bulletin of the Medical Library Association (BMLA)*, founded and maintained the MLA Exchange, and participated in other critical activities to keep our young association going and growing. No task was too small or beneath her.

And here is where my ghost story begins.

JMLA Journal of the Medical Library Association

SC

Supplemental content to
J Med Libr Assoc. Jan;105(1): dx.doi.org/10.5195/jmla.2017.127
www.mlanet.org
© Tooey 2017

SLIDE 15

Marcia Noyes was recruited, even hand-picked by Sir William Osler, to become the librarian at the Medical and Chirurgical Faculty of Maryland (MedChi) in 1896, 120 years ago. She knew absolutely nothing about medical librarianship but developed a comprehensive medical library that served as a resource library until the early 1990s, and learned on the job—early evidence of MLA experiential learning! By all accounts she was extremely competent and well liked, so much so that when the new MedChi building was opened in 1909, she was given an apartment in the building. It was said to be the first penthouse apartment in Baltimore [14–16].

SLIDE 16

She and Dr. Osler were lifelong friends until his death in 1919. She remained at MedChi for fifty years until her death in 1946. Another synchronistic moment—seventy years ago.

Even though I am a resident of Baltimore and knowledgeable about MedChi, I had forgotten about Marcia being there. So I reached out to Meg Fielding, MedChi's director of development, I arranged a visit. Sadly, the medical library is no longer in existence, having been closed in 2004. Some of the collection was auctioned off, some of it was taken by members, and the rest remains moldering in stacks with no heat or air conditioning. However, I was assured that Marcia was still there. Staff hear footsteps and computer keys clicking when alone in the building. If they are looking for something that is lost or something they need, things mysteriously appear. They believe in her ghost, although they are a little less respectful of her than we might be, decorating her digital image with costumes for different holidays. Bunny ears on Marcia? Really? No wonder she haunts them.

After a tour where I actually saw Marcia's once lovely apartment, now converted to offices, I jokingly said to Ms. Fielding that using the prism of today's society, one might wonder if Osler and Noyes were more than friends? As I entered my car, I looked back at MedChi, found her apartment on

Supplemental content to
J Med Libr Assoc. Jan;105(1): dx.doi.org/10.5195/jmla.2017.127
www.mlanet.org
© Tooey 2017

top of the building, and a giant plop of water landed on my head. It was not raining, and there was nothing above me. Marcia may have been being playful or perhaps cautioning me about being impertinent. Regardless, I believe.

SLIDE 17

I discovered she was buried at the Green Mount Cemetery, which is an old cemetery in Baltimore where John Wilkes Booth is buried along with Baltimore luminaries, famous and infamous. I contacted the cemetery to learn the location of her grave.

SLIDE 18

A few weeks ago, I visited her grave and left a bouquet to make amends for my impertinence. It is said she loved flowers, and for the past few years, MedChi has actually sent a bouquet to our Noyes Award winner. Call me crazy, but I had an internal conversation with her letting her know just how important she was and how far MLA had grown and thrived. I wanted to be right with Marcia.

But why was she our first woman, nonphysician president? She was eager to learn, eager to serve, instilled loyalty in her employees and her colleagues. Her first employer in Baltimore at the Enoch Pratt Free Library recommended her to Osler as "woman of executive talents." She believed in professionalizing medical librarianship, and she had a passion for that.

"Googling" "leadership qualities leads to a plethora of entries one could pursue forever. So, I turned to the library literature, where I found information from the New Members Round Table of the American Library Association [17].

Supplemental content to
J Med Libr Assoc. Jan;105(1): dx.doi.org/10.5195/jmla.2017.127
www.mlanet.org
© Tooey 2017

SLIDE 19

They have a comprehensive listing of leadership characteristics falling into seven categories: physical, emotional, social, intellectual/intelligence, communication, experience, and trustworthy. And while they do blur some of the lines between management and leadership, they break down each category further. We would recognize many of the terms they include as being standard to the current leadership literature. Terms such as agility, vision, risk taking, and self-awareness are touted as attributes for library leaders today. Would those leadership attributes be found in our past leaders?

SLIDE 20

All of us are probably familiar with MLA's oral histories, and if you are not, then you need to go back and read Barbara Epstein's 2015 Janet Doe Lecture, entitled "In Their Own Words: Oral Histories of Medical Library Association Past Presidents," in the January 2016 *JMLA* [18]. However, you may not be aware that for over five decades the *JMLA,* formerly the *BMLA,* has included presidential profiles to introduce you to new presidents.

Inspired by the 2012 Doe Lecture by Mark E. Funk, AHIP, FMLA, where by analyzing textual trends in our literature [19], he somehow compared the quantity of our output to the height of the Burj Khalifa in Dubai (I still think it is smoke and mirrors), I decided to try a text analysis.

And while my text analysis project of presidential profiles smacks more of Wiz Khalifa than the Burj Khalifa, it did yield some interesting results. But it was really hard to do a graphic with Wiz Khalifa and all our words, so I decided not to do it.

SLIDE 21

The text analysis software was applied to approximately 58 available presidential profiles, including recent profiles and ones further back. These profiles contained 64,373 words, and of those words, 7,640

Supplemental content to
J Med Libr Assoc. Jan;105(1): dx.doi.org/10.5195/jmla.2017.127
www.mlanet.org
© Tooey 2017

were unique. I deleted the 176 unique common words that accounted for 37,000 usages. Things like "a, an, the"—we use those a lot. There were about 27,000 words were left to analyze. There were still 7,464 unique words remaining. However, fully 3,888 of them were only used once. Initially, the results were quite discouraging, as not many of the most used words in the profiles were ones associated with leadership traits. Shouldn't our presidential profiles focus on leadership attributes? There were instances when current key leadership attributes such as agility or innovation were mentioned, but maybe only once or twice.

It is also important to note these are word occurrences and are not in context of or in relation to other words. Clearly, something had to be done. By combining similar terms and attributes, patterns started to develop and occurrences grew. The combinational categories were:

SLIDE 22

The "playing nice with others," category where I used about 10 terms including things such as cooperation, collaboration, partnerships, collegiality. And they were combined for about 45 occurrences in all those words.

Second, I looked at the management words: 22 terms were combined for 56 occurrences. And we used words such as practical, effective, capable, efficient—that kind of thing.

And then we looked the innovation traits: 20 terms combined for 75 occurrences. Things like vision, imagination, agility, innovation.

And one of my favorites, personal traits: humor, energy, stamina, passion, 51 terms combined for 212 occurrences.

Other areas that scored fairly well were terms associated with mentoring (44 occurrences), ethics (20 occurrences), and politics (18 occurrences.)

Supplemental content to
J Med Libr Assoc. Jan;105(1): dx.doi.org/10.5195/jmla.2017.127
www.mlanet.org
© Tooey 2017

And just to put all this in perspective, the term "MJ" scored 25 times, and "Tooey" scored 40 times for an overall combinational score of 65, putting me somewhere between innovation and management. Yesssss! It was kind of like Googling myself. I couldn't resist.

SLIDE 23

In leaving this exercise behind, there were many attributes that resonated with today's leadership language, such as vision, cooperation, practicality, passion, humor…and M.J. Tooey.

So while endlessly fascinating but incredibly time consuming, this was not the most illuminating way to assess the leadership attributes of our past leaders. One observation regarding the presidential profiles is after reading older ones and comparing them to more recent ones, the older ones tended to review professional accomplishments, while more recent profiles tell stories about the presidents including families, pets, favorite pastimes, hobbies, and, oh yes, those pesky professional accomplishments. My interpretation is the presidential profiles serve to humanize our leaders, introducing them to members who may not know them, rather than focusing on their leadership capabilities.

Since the analysis of mostly dead presidents and their profiles wasn't exactly satisfactory, the next step in the journey was to contact the living. Approaching the immediate past twenty-five years of MLA presidents was a joy, with an incredible response rate and a generosity of response.

SLIDE 24

Seven questions were sent to each former MLA president who served over the past 25 years. Two of the former presidents are deceased. Eighteen former presidents responded very generously, for a response rate of just over 78%.

JMLA Journal of the Medical Library Association

Supplemental content to
J Med Libr Assoc. Jan;105(1): dx.doi.org/10.5195/jmla.2017.127
www.mlanet.org
© Tooey 2017

Question 1: What was your path to MLA Leadership? Overwhelmingly, 100% of the answers to this question reflected experiential leadership in sections, chapters, and on committees and task forces.

Question 2: What leadership qualities did you bring to the MLA presidency? Many said they brought: Organizational skills. Knowledge of the profession. Vision. Enthusiasm. Communication skills. Partnering skills. Passion for and belief in what MLA does.

Question 3: While president, what was your greatest leadership challenge? Well, a large number responded that budget and financial issues were their greatest leadership challenges. Some were impatient with the slowness of progress. And some reflected on unforeseen challenges beyond their control such as the loss and closure of hospital libraries or illness, Hurricane Katrina, or the anthrax scare.

Question 4: What was the greatest lesson learned? Members are our greatest asset. Keep dreaming. Be selfless. Be compassionate. Politics are everywhere. Remember to thank people. Be flexible. Be opportunistic and take risks. And then finally, that they could actually do this thing called the presidency.

Question 5: Have the leadership qualities needed for the MLA presidency changed? Foundationally, no, but things have sped up. Social media has accelerated the way we need to react and communicate. Presidents need to be advocates and be able to forcefully articulate and advocate our vision. They need to be fearless. They need to have courage. They need to be creative. They need to be flexible. They need to be inventive. They need to be politically savvy and have the ability to create consensus and build partnerships.

By now, I had softened them up for the big question. Would their responses be significantly different from those I discovered in the presidential profiles?

Question 6: What is the most important trait for a leader to have? The ability to see the big picture. To have communication skills across technologies and interpersonally. To listen and be fully present. And to have compassion—and to remember everyone comes to the table with assets—and be

JMLA Journal of the Medical Library Association

Supplemental content to
J Med Libr Assoc. Jan;105(1): dx.doi.org/10.5195/jmla.2017.127
www.mlanet.org
© Tooey 2017

"fully appreciative of the blessing of leadership." Look broadly at the world around to see influences and trends. Integrity. Passion for the profession [10].

Question 7: Was there anything else they wanted to share? You have to add that in there as the last question. Of those who responded, almost 100% reflected a thankfulness for the opportunity to have been MLA president.

SLIDE 25

Common leadership words emerging from former presidents compared with the presidential profile analysis were concepts such as passion, (of course) having vision, and then articulating and advocating for our shared vision, integrity, communication skills, valuing partnerships, and collaboration. New terms included fearlessness, courage, creativity, flexibility, inventiveness, and political savviness. These newer terms modernized and expanded on many of the traits found in the presidential profile analysis and are evolutionary in nature. They may be very well reflective of the era in which we live and the greater personal access I had to these past presidents.

The next step in the leadership trait exploration journey was more granular and involved engaging the current presidential leadership triumvirate plus one in conversation. Following that conversation, I then repeated a study that I had done almost ten years ago with the Board of Directors.

SLIDE 26

The presidential triumvirate included Past President Linda Walton, AHIP, FMLA, President Michelle Kraft, AHIP, President-Elect Teresa L. Knott, AHIP, plus one more—the president elect-elect, the heir, and a spare, Barbara Epstein (remember this was during my time of trying to be nice to her—I wanted to make her feel included). There were no awkward pauses with this group!

Supplemental content to
J Med Libr Assoc. Jan;105(1): dx.doi.org/10.5195/jmla.2017.127
www.mlanet.org
© Tooey 2017

This wide-ranging discussion explored a number of issues. Their own particular strengths as leaders was one area we explored. Passion for the profession, there it is again, came up again and again along with an interest in people—engaging them, talking to them, learning from them, and sharing with them. When asked what qualities an MLA president needed, they responded to "wear armor" (I said, "This is not a leadership quality, and MLA is not *Game of Thrones*"), so that response became not to take things personally. Presidents need to leave their egos at the door. Know that sometimes their ideas are not the best ideas—be collaborative, approachable. Try to understand issues from a variety of perspectives—that's empathy—to be a consensus builder. Be a risk taker. Acknowledge the absurdity and ridiculousness of situations. Have fun. Have courage. Their conversation gave real substance to some of the traits I had identified in the presidential profiles and by the former presidents. Interestingly, their comments were very similar to the types of skills the literature says leaders will need in the future. More on that later. Their comments continued to move the leadership journey further down the road. The evolution of presidential traits seemed to show a need for connectivity to the membership and beyond. Interpersonal qualities have evolved to take on more importance.

In 2006/07, as past-president, I chaired the MLA Nominating Committee. In order to give some guidance to the committee, I surveyed the MLA Board at the time regarding leadership qualities presidents should have. I thought, "Well, they're the ones who work with the president, they would be good folks to ask what they think a president should be like."

SLIDE 27

In 2006, this is what the MLA Board said were the key attributes for the president: And you can see the comparison.

1. They need to be a visionary with the ability to build shared vision/consensus among disparate communities.

Supplemental content to
J Med Libr Assoc. Jan;105(1): dx.doi.org/10.5195/jmla.2017.127
www.mlanet.org
© Tooey 2017

2. They need to be a good communicator—articulate, and an articulator of vision, a good listener and practitioner of two-way communication.
3. They need to have broad background and knowledge of MLA and professional issues affecting members and our environment and have the ability to represent our association and profession to others.
4. They need to have the ability to motivate, persuade, inspire, and mobilize individual commitment.
5. They need to encourage collaboration.
6. They need to have enthusiasm and energy with physical and emotional stamina.
7. They need to have respect for colleagues throughout the association with a connection with all members (in many different ways—appropriate to need and audience), not just with their type or within their comfort zone and have an interest in active engagement.
8. They need to be open to new people/ideas.
9. They need political agility and awareness.
10. They need humility.
11. They need comfort and grace in the spotlight.
12. And they need a passion for the profession.

In 2016, this is what the board members had to say. The president needs to be:

1. They need to be a good communicator including being virtually savvy and being a good listener.
2. They need to be a visionary.
3. They need interpersonal skills.
4. They need to be self-confident.
5. They need to have enthusiasm, energy, stamina. Stamina kept coming up—that was interesting.
6. They need to be calm under pressure.
7. Trustworthy, respectful, patient, humble.

JMLA Journal of the Medical Library Association

Supplemental content to
J Med Libr Assoc. Jan;105(1): dx.doi.org/10.5195/jmla.2017.127
www.mlanet.org
© Tooey 2017

8. Good sense of humor,
9. Organizational and problem-solving skills.
10. Past leadership experience.
11. Passion for MLA.

With ten years between surveys, the top responses, albeit in different order or phrasing, were very similar as you can see: a good communicator, a visionary, a consensus builder, politically savvy, someone who is emotionally intelligent and sensitive to, and respectful of, all MLA members. Stamina, humor, and humility. And let's not forget being passionate about our profession.

It is tempting to say we expect our leaders to walk on water. However, are these leadership attributes any different than what was already discovered among all the leaders I reviewed? The attributes of past presidents, the presidential triumvirate plus one, and the board members align well with the attributes from the text analysis of the presidential profiles with, of course, new attributes being added in for our time.

SLIDE 28

Would it surprise you to know there was one more conversation? This is the stamina part—you keep talking to people. This consisted of talking to the current and former executive directors of MLA: Kevin Baliozian and Carla J. Funk, CAE.

Their outside but inside perspective on leadership added nuances to the leadership qualities of MLA presidents.

Here is what I learned from them with this question: What were the leadership attributes of successful MLA presidents?

JMLA Journal of the Medical Library Association

Supplemental content to
J Med Libr Assoc. Jan;105(1): dx.doi.org/10.5195/jmla.2017.127
www.mlanet.org
© Tooey 2017

They felt the most successful MLA leaders they knew were cheerleaders; they were facilitators, not-ego driven, humble; they encouraged different viewpoints. They engaged the members and were inclusive. They were someone who saw the big picture, yet focused on the work to be done.

Please note, and I think this is important because we hear this a lot in our profession, that with the exception of one comment from a former president who said it didn't matter, not one person commented on the desirability of being an extrovert or an introvert. There is plenty in the literature regarding pros and cons of those personality traits in leaders, and entire leadership lectures could be done on the topic. I would like us going forward to consider the term "ambivert" [20]. It's a term, search it! A person who exhibits qualities of both extroversion and introversion, as is needed in the situation. Increasingly, this term is appearing in the literature as a desirable leadership trait [21, 22].

SLIDE 29

At this point, we have a good, fairly uniform picture of what MLA's leaders across our distant and near history have identified as critical leadership traits. In a summary analysis, many traits are similar. The more contemporary responses are in line with, and reflect, personal attributes very common to the current leadership literature…leadership traits with a healthy dose of management thrown in, focusing on self-awareness, people skills, and emotional intelligence. I surmise that if we had the opportunity to survey or converse with Marcia Noyes, and I may have that opportunity if she appears as a ghost, or other of our past MLA leaders who don't have oral histories, we would discover further alignment and similarities. But all we have are presidential profiles, letters, minutes, and perhaps an oral history to reveal these qualities. And so after 64,373 words in 58 profiles, 23 past presidential surveys, conversations with current presidents, and past and current executive directors, and 2 board surveys, what did I learn?

Our enduring leadership traits are: exemplary communication skills in all ways and directions, vision, collaboration, and consensus building skills. Our presidents, our heroes need to have

JMLA Journal of the Medical Library Association

Supplemental content to
J Med Libr Assoc. Jan;105(1): dx.doi.org/10.5195/jmla.2017.127
www.mlanet.org
© Tooey 2017

energy/stamina, humility, and my personal favorite—humor—continue to provide a foundation for our leaders. And the greatest commonality? Overwhelmingly, a passion for our profession. Simply, you have to love what you do.

Let us shift the perspective a bit and look at what this means not only for leaders in our association and profession, but to us personally. What does the literature say will be critical attributes of future leaders? As our professional home, what is MLA's responsibility, and what is our own personal responsibility to ensure we are preparing engaged, successful leaders not only for our libraries, whatever that means or whatever they become, but for institutions in a landscape where change is constant and pervasive? It is not enough to look at the changes in the health sciences and health care because our future leaders will be called upon to navigate changes in technology, higher education, our communities, emerging diseases, and globalization areas, using skills to connect and bridge knowledge and information chasms. In a society where information is ubiquitous, constant, and accessible, what will be our role? What will our leaders look like? What will all of you look like? What traits will you need to grow and mature in new roles we haven't even imagined yet? Many of the traits—such as agility, self-awareness, good communication skills in all forms and formats, vision, and passion—we have already identified, and according to the literature, they will remain relevant.

What are some additional traits to be considered?

SLIDE 30

We need to consider:

Risk taking: Although this was mentioned several times, risk taking, although not a leadership quality, is actually a process, not a leadership trait. Risk takers pore over details and study situations; they know the challenges and objectives inherent in taking a risk. We must become comfortable with that process [23].

Supplemental content to
J Med Libr Assoc. Jan;105(1): dx.doi.org/10.5195/jmla.2017.127
www.mlanet.org
© Tooey 2017

We need to have an innovative mind set. We need to look at opportunities in new and different ways, finding new solutions and perhaps taking same risks.

We need to embrace, accept, and cause disruption: Disruption simply creates a new normal. In order to embrace disruption, leaders need to pursue the truth, be willing to guide others through chaos, make decisions; they break the rules and write new ones but always with an explanation of why; and they thrive on uncertainty [24].

We need to develop a tolerance for failure: Failure doesn't mean that you're done, it just means you need to reassess and recalibrate—you may fall down seven times but you need to get up eight [24].

Resilience: Andrew Zolli, a former MLA keynote speaker a few years back wrote in his book, entitled "Resilience: Why Things Bounce Back," that resilience is the capacity of a system, enterprise, or person to maintain its core purpose and integrity in the face of dramatically changed circumstances [25].

We need to be empathic: Being able to understand another's viewpoint and put oneself in their shoes and in that situation. It is not sympathy.

Develop support networks: mentors, colleagues, and buddies. I call them frolleagues (friend colleagues).

Warren Bennis believed leaders are made, not born. He compared leadership to a performance where leaders must inhabit their roles as actors do—not only learning to see themselves as others do, but also through a process of self-discovery. In 2009, he said that "the process of becoming a leader is similar, if not identical to becoming a fully integrated human being" [5].

SLIDE 31

How does MLA light that spark and support risk takers, innovators, disruptors, failures, empaths, and "frolleagues." How do we cultivate, and grow and encourage leaders to, as Bennis said, "inhabit" these leadership roles? [26]

Supplemental content to
J Med Libr Assoc. Jan;105(1): dx.doi.org/10.5195/jmla.2017.127
www.mlanet.org
© Tooey 2017

Throughout my years as a member of MLA, there has been an increasing commitment to leadership development. This seemed to begin in the mid to late 1990s, perhaps as MLA's leadership realized that within a decade, a lot of members would begin retiring. New methods of communication and information sharing required new ways to look at leadership. It became easier to collaborate and share ideas. The Internet, email, an electronic *JMLA,* the rise of consumerism and exposure to new ideas, and the increasing ability to engage members changed the leadership paradigm from one of our leader being the all-knowing head of MLA to one of chief collaborator, mentor, and servant leader.

MLA's strong continuing education (CE) program has supported courses, webinars, and symposia on leadership. In May of 2002, a symposium, entitled "Leadership Reconsidered: Developing a Strategic Agenda for Leadership in Health Sciences Libraries" [27]. The following recommendations were made:

1. Offer a CE course on being a director.
2. Scholarships for educational opportunities.
3. Host a leadership institute.
4. Support a "chatroom" for the discussion of leadership issues. There's an old word, chatroom.
5. Classes or CE opportunities on political skills in whatever format is appropriate.
6. Encourage chapter and section leadership opportunities.
7. Journal club on leadership.
8. Provide pathfinders of resources related to leadership.

Many of these recommendations from 2002 have come to fruition. Our already strong CE program now includes at least four courses on leadership. There is a move toward developing a webinar series focused specifically on leadership traits and needs.

I am particularly excited about an educational initiative called the Nexus Project, which is being funded by the Institute for Museum and Library Services, that will be available in 2017 [28]. Carol G.

Supplemental content to
J Med Libr Assoc. Jan;105(1): dx.doi.org/10.5195/jmla.2017.127
www.mlanet.org
© Tooey 2017

Jenkins, AHIP, FMLA, a long-time leader in our profession and an advocate for leadership development, and Carla Funk, the former MLA executive director, are part of the development team.

This project addresses six different levels of leadership: Leading Self, Leading Others, Leading the Department, Leading Multiple Departments, Leading the Organization, and Leading the Profession. Within each level, there are daily challenges and different things that need to be done. When it is ready, it promises to stimulate much discussion.

Now MLA's awards program recognizes leaders in hospital libraries, researchers, young leaders, and experienced leaders.

The Estelle Brodman Award for Academic Medical Librarian of the Year honors an academic librarian at mid-career. Winning the award in 1997 was an affirmation of the work I was doing and the path I had chosen. It was an incentive to keep going with MLA. Once a long time ago, I jokingly mentioned to Carla Funk that if I ever got to do the Doe Lecture (duh, duh, duh—that's the foreshadowing music), my presentation would be "Brodman Award Winners: Did They Live Up to Their Potential?" After a scathing, withering look from Carla and an "Oh MJ!," I dropped that idea. See what bullet you dodged? But I have continued to wonder about the effect of winning the award on the recipients.

Would it surprise you to know I surveyed former Brodman Award winners and asked that very question? Hey, when you are the Doe lecturer, you have access to a lot of stuff. There have been 25 winners since it was established in 1988. Two award winners are deceased and many are retired. Some have left the profession. Within the 14 responses received (61% response rate), most responded in the same way: it had been an honor. It reinforced connections to MLA. Most stayed involved, and most continued their leadership journeys right into retirement. But some did articulate the pressure to live up to the award and expectations of their professional performance. Bart Ragon, scoundrel, the 2012 Brodman awardee and I have been discussing how the Brodman Award winners might work together to

Supplemental content to
J Med Libr Assoc. Jan;105(1): dx.doi.org/10.5195/jmla.2017.127
www.mlanet.org
© Tooey 2017

"pay it forward" so to speak and support the development of mid-career librarians. As a group of people who have been acknowledged as mid-career academic librarians with potential, whether we lived up to it or not, how can the Brodman awardees leverage our experience, and support, not only academic librarians, but all library colleagues who need to "lead from the middle?" How do we encourage advancement for those who aspire to become directors? How do we engage those whose strength, talent, and passion IS leadership in the middle? How do we act as catalysts and mentors? We have a Facebook page; it is called the Brodman Page, is open to everyone, and we would love to hear any ideas. A small group of us will be getting together later today here at MLA to begin the discussions.

Does being involved in leadership programs, winning an award, or taking a leadership course make a person a leader? Of course not, but it provides opportunities for exploration, self-awareness, determining leadership passions, examining personal leadership journeys and readiness, and most of all, your passion for leadership. Being a leader is not for the faint of heart—it takes courage.

But perhaps the most valuable leadership development tool MLA has are its twenty-one sections, twenty-five special interest groups (SIGs), thirteen chapters, and lots of committees and task forces. As a firm believer in experiential leadership, there is no substitute for the experience of doing. Leadership needs to be nurtured and cultivated, and our chapters, sections, committees, and task forces are MLA's personal incubator. Sometimes, the situation creates the leader. Think of Winston Churchill and World War II or Franklin Delano Roosevelt and the Great Depression.

MLA actually has a Leadership and Management Section, which was the Medical School Libraries Section until the early 2000s, when section leaders realized there was an important need to be met in encouraging leadership development. Remember that 100% of the most recent 25 years of MLA presidential respondents gained their leadership experience through sections, chapters, committees, or task forces. MLA must continue to find ways to leverage leadership experience within these valuable leadership training grounds. While it is not a section, chapter, committee, or task force, the Rising Stars

JMLA Journal of the Medical Library Association

Supplemental content to
J Med Libr Assoc. Jan;105(1): dx.doi.org/10.5195/jmla.2017.127
www.mlanet.org
© Tooey 2017

program, where aspiring leaders are selected and assigned to an MLA-based project, is a great example of the way MLA is providing a leadership experience within the context of the work of the association. Leadership in context—similar to an informationist…a leadernist? I joke.

And I would say to those of you who were leaders or are leaders, your work is not done. You need to continue to engage with our emerging and aspirational leaders as mentors and coaches.

SLIDE 32

In "The Five Practices of Exemplary Leadership" by James Kouzes and Barry Posner [29], they ask that leaders do five things; they ask them to:

- Model the way
- Inspire a shared vision
- Challenge the process
- Enable others to act
- Encourage the heart

It is "encouraging the heart" that draws me toward the end of the 2016 Janet Doe Lecture. This will probably what I will spend the least amount of time on today but is perhaps of most importance to me. And it is where I want to leave us today.

SLIDE 33

And that is with the concept of the small "l" lower case leadership. I viewed over two dozen TED Talks in preparation for today.

Supplemental content to
J Med Libr Assoc. Jan;105(1): dx.doi.org/10.5195/jmla.2017.127
www.mlanet.org
© Tooey 2017

SLIDE 34

By far the most inspiring TED talk I viewed was by Drew Dudley, a leadership educator, who gave his talk in 2010 right here in Toronto at the Toronto TEDx, entitled "Everyday Leadership" [30]. I would ask all of you to think about this—and I quote Drew, "How many of you are uncomfortable with calling yourself 'leaders?' We don't let ourselves take credit for it. Treating it is as something someday we may deserve." He continues, "And I've come to realize that we have made leadership into something bigger than us. We've made into something beyond us. We've made it about changing the world. And we've taken this title of leader, and we treat it as if it's something that one day we're going to deserve, but to give it to ourselves right now means a level of arrogance or cockiness that we're not comfortable with."

I believe his point is that we need to get over that. We need to value the impact we have on each other's lives and that we need to redefine leadership because, and I continue to quote, "we've made leadership about changing the world and there is no world. There's only six billion understandings of it, and if you change one person's understanding of it, one person's understanding of what they're capable of, one person's understanding of how much people care about them, one person's understanding of how powerful an agent for change they can be in this world, you've changed the whole thing. And if we can understand leadership like that, if we can redefine leadership like that, I think we can change everything."

Let's try an exercise. You've been sitting for a long time, and I appreciate that. I am going to ask you to stand. Actually sit now and then stand as I call your name. I'm sorry—not good direction on my part—this is why I am not a teacher. I don't know when I am going to do these things.

How many of you have ever been Doe lecturers, won the Marcia C. Noyes Award or any other MLA award? Stand up. Okay. Very good

How many of you have served as president or on the Board of Directors? More standing. Okay.

Supplemental content to
J Med Libr Assoc. Jan;105(1): dx.doi.org/10.5195/jmla.2017.127
www.mlanet.org
© Tooey 2017

How many of you have served on an MLA committee or task force? Very good. It's getting bigger.

Have served as a chapter or section officer or on a chapter or section committee? There should be more people standing.

Are members of sections or chapters?

Here's the trick question. Have attended an annual meeting of MLA? Up. Up. Up. Yeah, you have to stand, Kevin. Get up.

Look around—look how many of you there are!

Every one of you is here because you have a commitment to your profession and to yourself. You are leaders. Sit down—otherwise it would look like I was co-opting a standing ovation

SLIDE 35

Your biggest impact may be something you may never remember doing or saying, that leads a change. That is leadership with a small "l."

When I have been in leadership positions, I have occasionally been frustrated by people expecting me to do the work, right a wrong, advance a position, advocate for justice. What I have come to realize is that people are simply looking for leadership. Most times, that leadership can be found within ourselves. We are all we've got.

Kouzes and Posner ask us to "inspire the heart." We all have the ability to inspire the heart.

I wish I could give you this next quote in the great Irish/English accent that the poet David Whyte has. The poet David Whyte asks us to "find that edge between its (your) own particular signature and genius and what it is being called into by its surrounding world." "You change the world by meeting it." "You change the world by meeting it" [31, 32].

JMLA Journal of the Medical Library Association

Supplemental content to
J Med Libr Assoc. Jan;105(1): dx.doi.org/10.5195/jmla.2017.127
www.mlanet.org
© Tooey 2017

Medical librarianship is in a time of tremendous change, and every one of us is needed now more than ever to lead and grow our profession, and our association, and ourselves. We need to "meet" our world. We can't be afraid to lead and start on our leadership journey with that one step.

We ALL can be heroes just for one day, one meeting, one moment, one comment, one conversation, one connection. And if we expect to advance, to thrive, and lead…it is not optional—we must all be heroes!

SLIDE 36

Thank you.

REFERENCES

1. Matheson NW. The idea of the library in the twenty-first century [Doe lecture, 16 May 1994]. Bull Med Libr Assoc. 1995 Jan;83(1):1–7. (Available from: <http://www.ncbi.nlm.nih.gov/pmc/articles/PMC225988/>. [cited 12 Dec 2016].)
2. Rothman J. Shut up and sit down: why the leadership industry rules [A Critic at Large]. New Yorker [Internet]. 29 Feb 2016 [cited 25 Jul 2016]. <http://www.newyorker.com/magazine/2016/02/29/our-dangerous-leadership-obsession>.
3. Brainy Quotes. Lao Tzu quotes [Internet]. [cited 25 Jul 2016]. <http://www.brainyquote.com/quotes/quotes/l/laotzu137141.html>.
4. Kotter JP. What leaders really do. Harvard Business Rev. 2001 79;(11):85–96.
5. Brainy Quotes. Warren Bennis quotes [Internet]. [cited 25 Jul 2016]. <http://www.brainyquote.com/quotes/quotes/w/warrenbenn385287.html>.
6. Basler TG. There are no more giants: changing leadership for changing times [Doe lecture, 2008]. Personal communication: Slides shared with Tooey MJ. Feb 2016.
7. Anderson RK. Reinventing the medical librarian [Doe lecture, 22 May1989]. Bull Med Libr Assoc. 1989 Oct;77(4):323–31. (Available from: <http://www.ncbi.nlm.nih.gov/pmc/articles/PMC227480/>. [cited 12 Dec 2016].)
8. Lipscomb CE. Race and librarianship: part I [historical notes]. J Med Libr Assoc. 2004 Jul;92(3):299–301. (Available from: <http://www.ncbi.nlm.nih.gov/pmc/articles/PMC442167/>. [cited 12 Dec 2016].)
9. Lipscomb CE. Race and librarianship: part II [historical notes]. J Med Libr Assoc. 2005 Jul;93(3):308–10. (Available from: <http://www.ncbi.nlm.nih.gov/pmc/articles/PMC1175796/>. [cited 12 Dec 2016].)
10. Homan JM. Inaugural address: promoting a passion for the profession—keeping our eyes on the prize! In: Proceedings of the One Hundredth Annual Meeting, Medical Library Association, Inc., Vancouver, British Columbia, Canada, May 5–11, 2000. Bull Med Libr Assoc. 2001 Jan;89(1):117–21. (Available from: <http://www.ncbi.nlm.nih.gov/pmc/articles/PMC31720/>. [cited 12 Dec 2016].)
11. Groen FK. Three who made an association: I. Sir William Osler, 1849–1919 II. George Milbry Gould, 1848–1922 III. Margaret Ridley Charlton, 1858–1931 and the founding of the Medical Library Association, Philadelphia, 1898. Bull Med Libr Assoc. 1996 Jul;84(3):311–9. (Available from: <http://www.ncbi.nlm.nih.gov/pmc/articles/PMC226151/>. [cited 12 Dec 2016].)
12. Smith BT. Marcia Crocker Noyes, medical librarian: the shaping of a career. Bull Med Libr Assoc. 1974 Jul;62(3):314–24. (Available from: <http://www.ncbi.nlm.nih.gov/pmc/articles/PMC198800/>. [cited 12 Dec 2016].)

Supplemental content to
J Med Libr Assoc. Jan;105(1): dx.doi.org/10.5195/jmla.2017.127
www.mlanet.org
© Tooey 2017

13. Marcia C. Noyes, medical librarian [obituary]. Bull Med Libr Assoc. 1947 Jan;35(1):108–9. (Available from: <https://www.ncbi.nlm.nih.gov/pmc/articles/PMC194645/>. [cited 12 Dec 2016].)

14. Harman SE. Marcia Noyes' correspondence: a life and profession reflected in letters. Md Med J. 1996 Jul;45(7):571–5.

15. Harman SE. Marcia C. Noyes: making a life. Md Med J. 1991;40(4):311–3.

16. Jensen JE. MedChi's diligent ghost. Md Med J. 1985;34(3):241–3.

17. American Library Association, New Members Round Table. Leadership traits [Internet]. The Association [cited 25 Jul 2016]. <http://www.ala.org/nmrt/initiatives/ladders/traits/traits>.

18. Epstein BA. In their own words: oral histories of Medical Library Association past presidents [Doe lecture, 18 May 2015]. J Med Libr Assoc. 2016 Jan;104(1):3–14. DOI: http://dx.doi.org/10.3163/1536-5050.104.1.002.

19. Funk ME. Our words, our story: a textual analysis of articles published in the Bulletin of the Medical Library Association/Journal of the Medical Library Association from 1961 to 2010 [Doe lecture, 21 May 2012]. J Med Libr Assoc 2013 Jan;101(1):12–20. DOI: http://dx.doi.org/10.3163/1536-5050.101.1.003.

20. Dictionary.com. Ambivert [Internet]. [cited 25 Jul 2016]. <http://www.dictionary.com/browse/ambivert>.

21. Vozza S. Forget introverts versus extrovert, ambiverts might have an edge in business. Fast Company [Internet]. 29 Feb 2016 [cited 25 Jul 2016]. <http://www.fastcompany.com/3057091/how-to-be-a-success-at-everything/forget-introverts-vs-extroverts-ambiverts-might-have-an-ed>.

22. Bradberry T. Nine signs that you're an ambivert. Forbes [Internet]. 24 Apr 2016 [cited 25 Jul 2016]. <http://www.forbes.com/sites/travisbradberry/2016/04/26/9-signs-that-youre-an-ambivert/#dfbc62243a61>.

23. Moran G. The surprising habits of the biggest risk takers. Fast Company [Internet]. 26 Feb 2016 [cited 25 Jul 2016]. <http://www.fastcompany.com/3057086/lessons-learned/the-surprising-habits-of-the-biggest-risk-takers>.

24. Hoque F. Five habits of truly disruptive leaders. Fast Company [Internet]. 9 Nov 2015 [cited 25 Jul 2016]. <http://www.fastcompany.com/3052725/hit-the-ground-running/5-habits-of-truly-disruptive-leaders>.

25. Zolli A, Healy AM. Resilience: why things bounce back. New York, NY: Simon and Schuster; 2012.

26. Schumpeter. Leading light. The Economist. 2014 Aug 9:57.

27. Martin ER, Brown HA, Epstein BA, Lipscomb CE, Riley RA, Shipman JP. The Leadership Reconsidered symposium: report. J Med Libr Assoc. 2003 Apr;91(2):251–7. (Available from: <http://www.ncbi.nlm.nih.gov/pmc/articles/PMC153168/>. [cited 12 Dec 2016].)

28. Educopia Institute. Nexus LAB: leading across boundaries [Internet]. The Institute [cited 29 Jul 2016]. <https://educopia.org/research/grants/nexus-lab-leading-across-boundaries>.

29. Kouzes JM, Posner Z. The five practices of exemplary leadership. 2nd ed. San Francisco, CA: Pfeiffer; 2011.

30. Dudley D. Everyday leadership [Internet]. TEDx Toronto; Feb 2010 [cited 12 Dec 2016]. <https://www.ted.com/talks/drew_dudley_everyday_leadership?language=en>.

31. Whyte D. Life at the frontier: the conversational nature of reality. TEDxPugetSound; 26 Feb 2011 [cited 12 Dec 2016]. <https://www.youtube.com/watch?v=5Ss1HuA1hIk>.

32. Tippett K, Whyte D. The conversational nature of reality. On Being [Internet]. [cited 7 Apr 2016]. <http://www.onbeing.org/program/david-whyte-the-conversational-nature-of-reality/8560/audio?embed=1>.
