# Supplementary material for: We can be heroes: MLA’s leadership journey(s)
Source: J Med Libr Assoc. 2017 Jan;105(1):88. doi: 10.5195/jmla.2017.127 (PMC5234446; doi:10.5195/jmla.2017.127)
Supplement: Appendix B [file jmla_jan17_tooey_appb.pdf]

JMLA Journal of the Medical Library Association
Supplemental content to
J Med Libr Assoc. Jan;105(1): dx.doi.org/10.5195/jmla.2017.127
www.mlanet.org
© Tooey 2017

We Can Be Heroes: MLA’s Leadership Journey(s)

Barbara and Me – the 80s

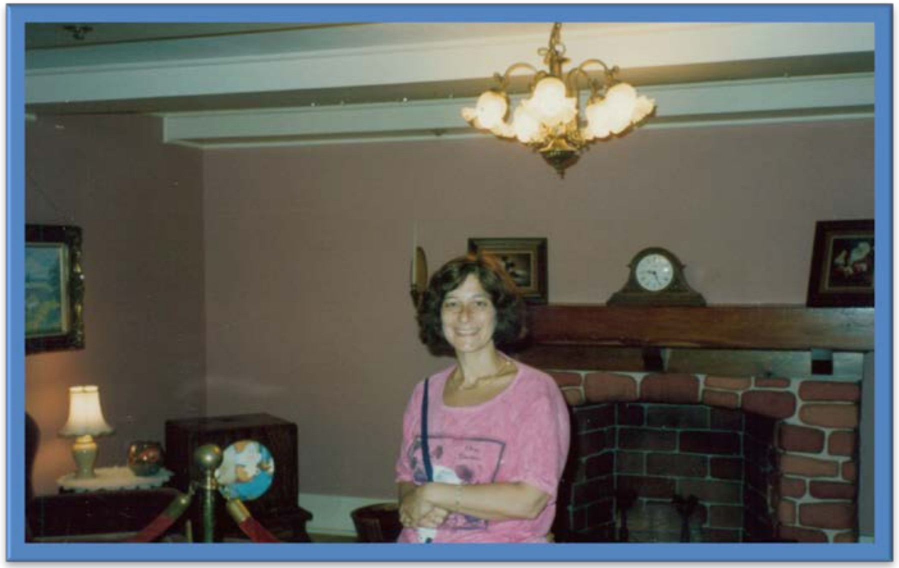

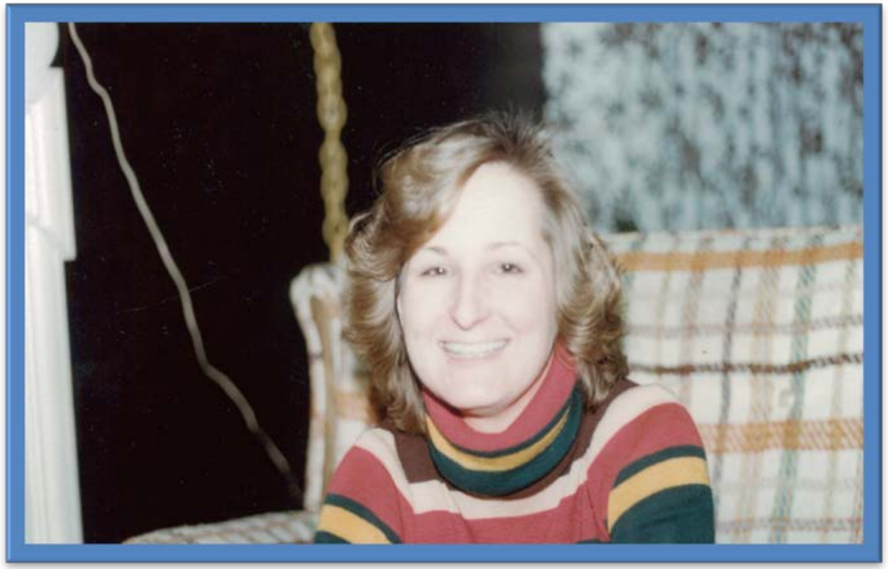

Thanks

- Kris Alpi
- Rachael Anderson
- Gretchen Arnold
- Kevin Baliozian
- Charles Bandy
- Margaret Bandy
- Tom Basler
- Amy Blevins
- Aphrodite Bodycomb
- David Bowie
- Estelle Brodman
- Naomi Broering
- Judy Burnham
- Karen Butter
- Margaret Charlton
- Kate Corcoran
- Janet Doe
- Jacque Doyle
- Barbara Epstein
- Julia Esparza
- Sandra Franklin
- Meg Fielding
- Rick Forsman
- Gary Freiburger
- June Fulton
- Stephanie Fulton
- Carla Funk
- Mark Funk
- Brad Gerhart
- Frances Groen
- Gale Hannigan
- Heidi Heilemann
- Patricia Hinegardner
- Heather Holmes
- Ruth Holst
- J. Michael Homan
- Bruce Jarrell, MD
- Carol Jenkins
- Diane Johnson
- Dixie Jones
- Bohyun Kim
- Robin Klein
- Teresa Knott
- Michelle Kraft
- Jan LaBeause
- Mary Langman
- Maria Lopez
- Nancy Lorenzi
- Richard Lucier
- Elizabeth Lund
- Alexa Mayo
- Nina Matheson
- Phyllis Mirsky
- Ray Naegele
- Marcia C. Noyes
- Jay Perman, MD
- Jerry Perry
- Jodi Philbrick
- Thom Pinho
- T. Scott Plutchak
- J. Dale Prince
- Bart Ragon
- Carolyn Reid
- Fred Roper
- Mary Ryan
- Charles Sargent
- Connie Schardt
- Chris Shaffer
- Jean Shipman
- Julia Sollenberger
- Nancy Tannery
- Pat Thibodeau
- Lisa Traditi
- UM HS/HSL Library Team
- Linda Walton
- Linda Watson
-

Special Thanks and Love

- Ron Huffman
- Greer Huffman Griffith

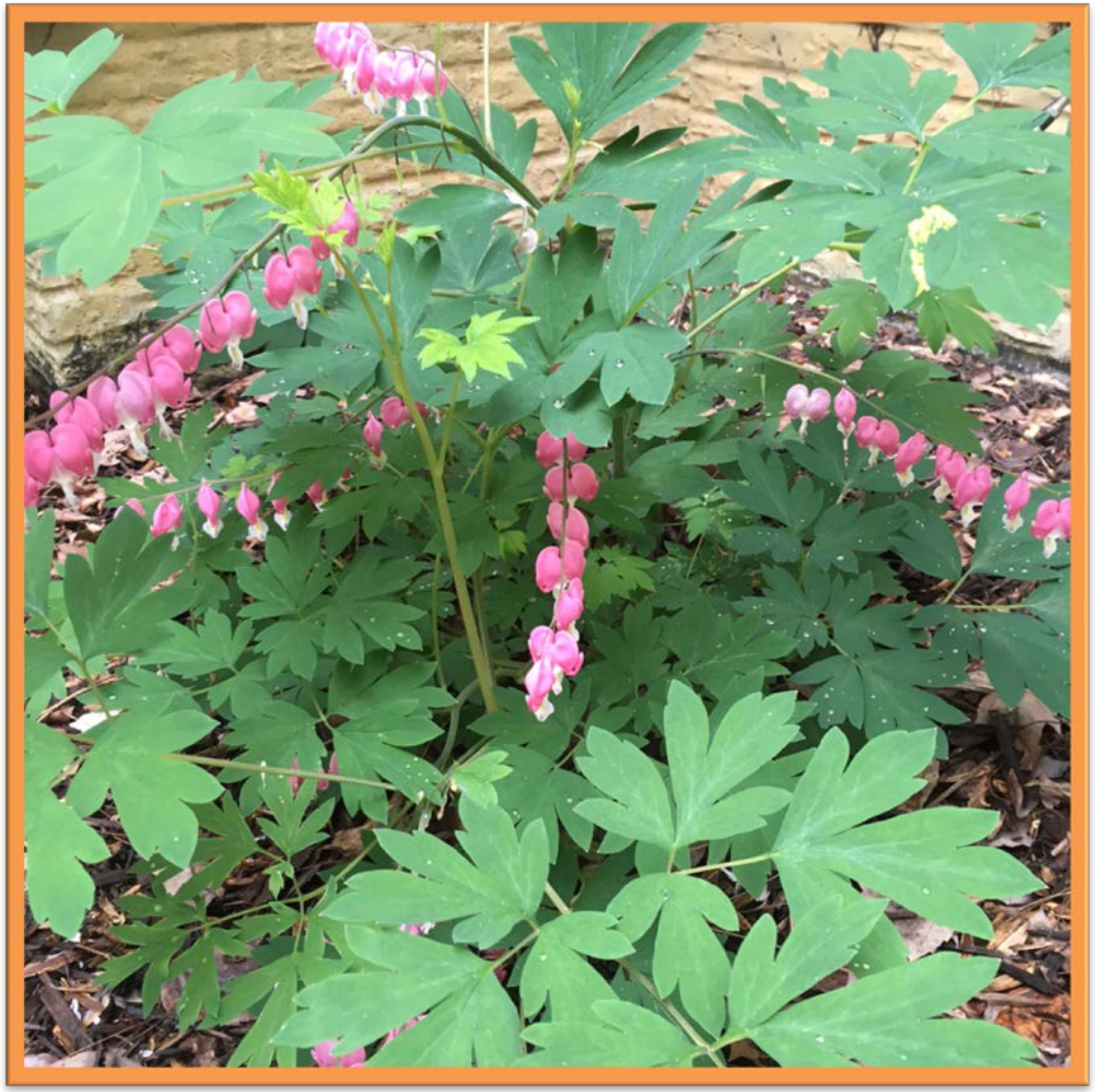

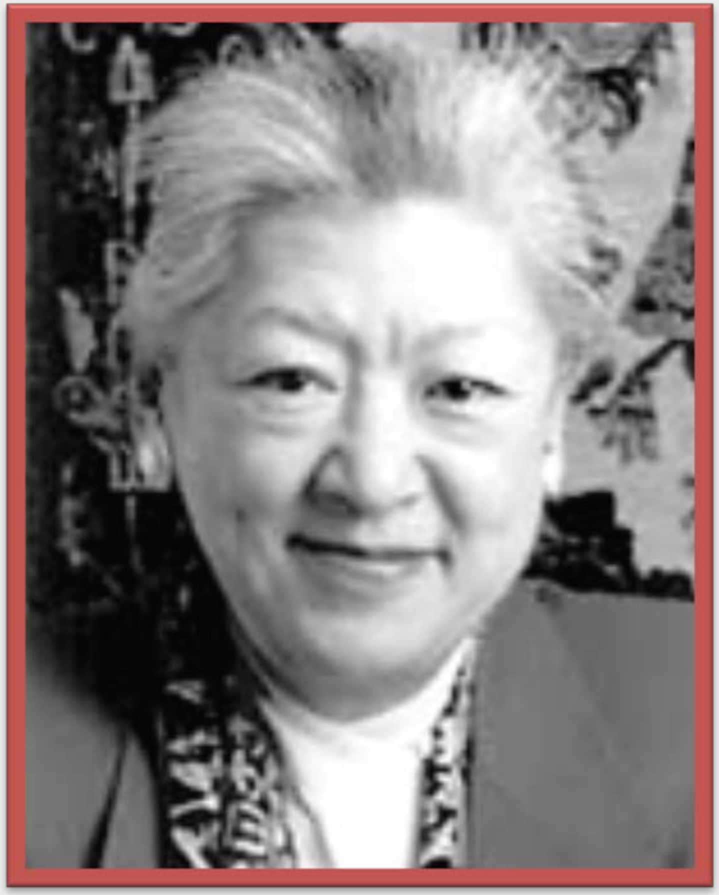

Nina Matheson – 1994 Janet Doe Lecturer

"The Idea of the Library in the 21st Century"

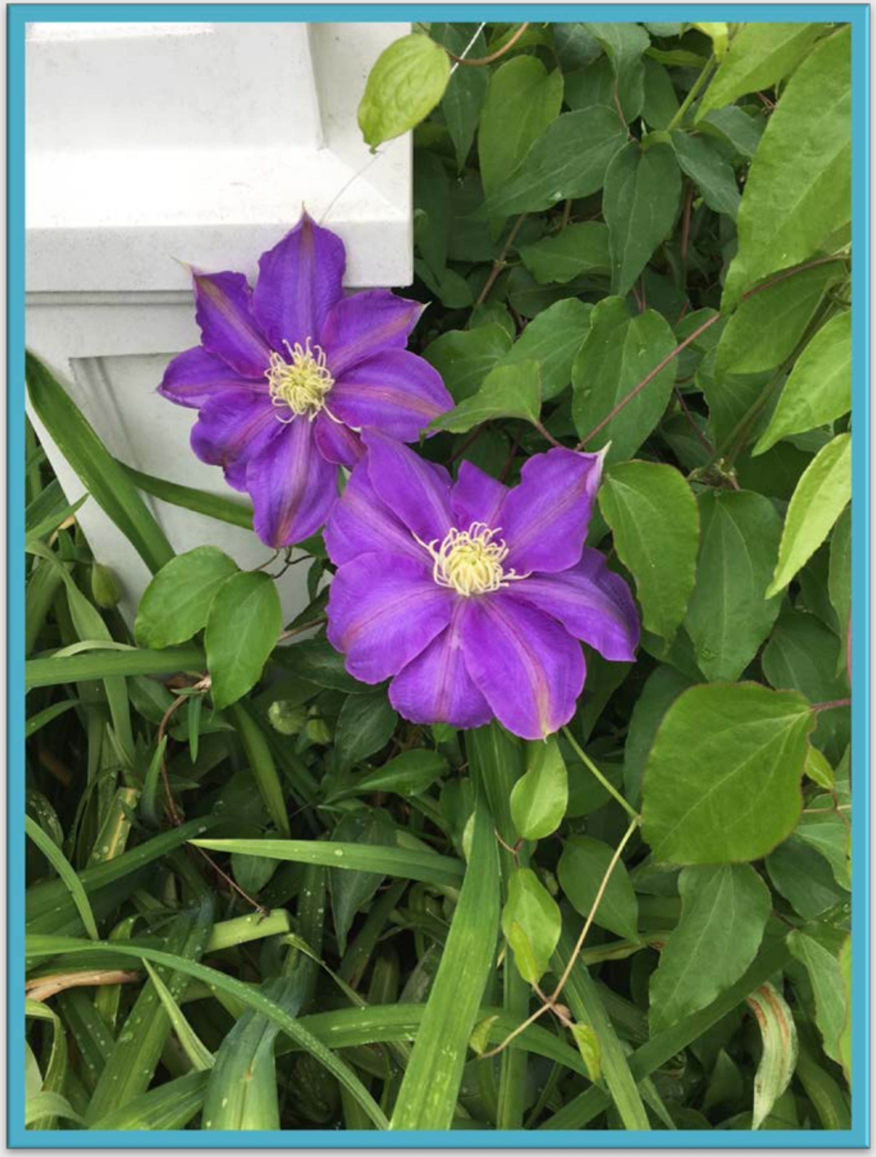

Truths and Confessions

1. No luminaries
2. No Does
3. No frills

50

30

10

David Bowie dies

(January 10, 2016)

We Can Be Heroes:
MLA’s Leadership Journey(s)

Kotter – "What Leaders Really Do"

Managers

- Planning/budgeting
- Organizing/staffing
- Controlling

Leaders

- Vision/strategies
- Aligning
- Motivating

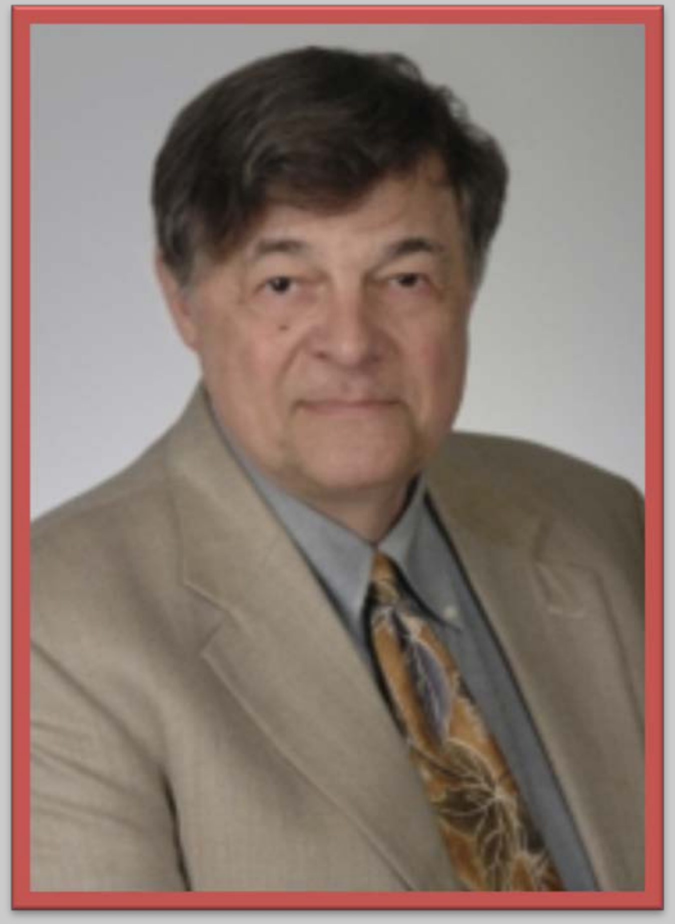

Tom Basler – 2008 Janet Doe Lecturer

"There Are No More Giants:
Changing Leadership for Changing Times"

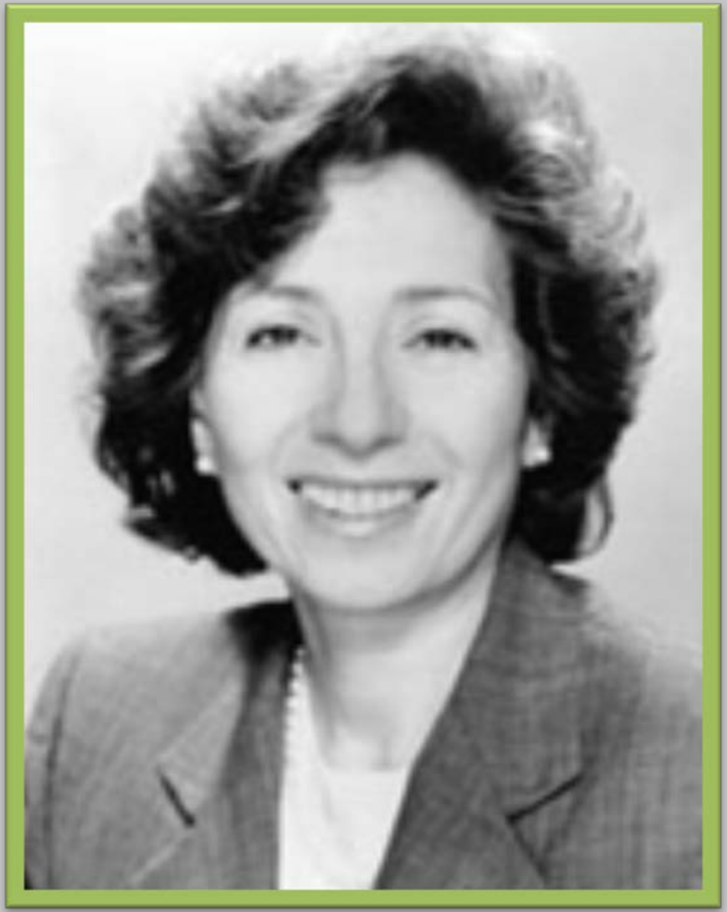

Rachael K. Anderson - 1989 Janet Doe Lecture

"Reinventing the Medical Librarian"

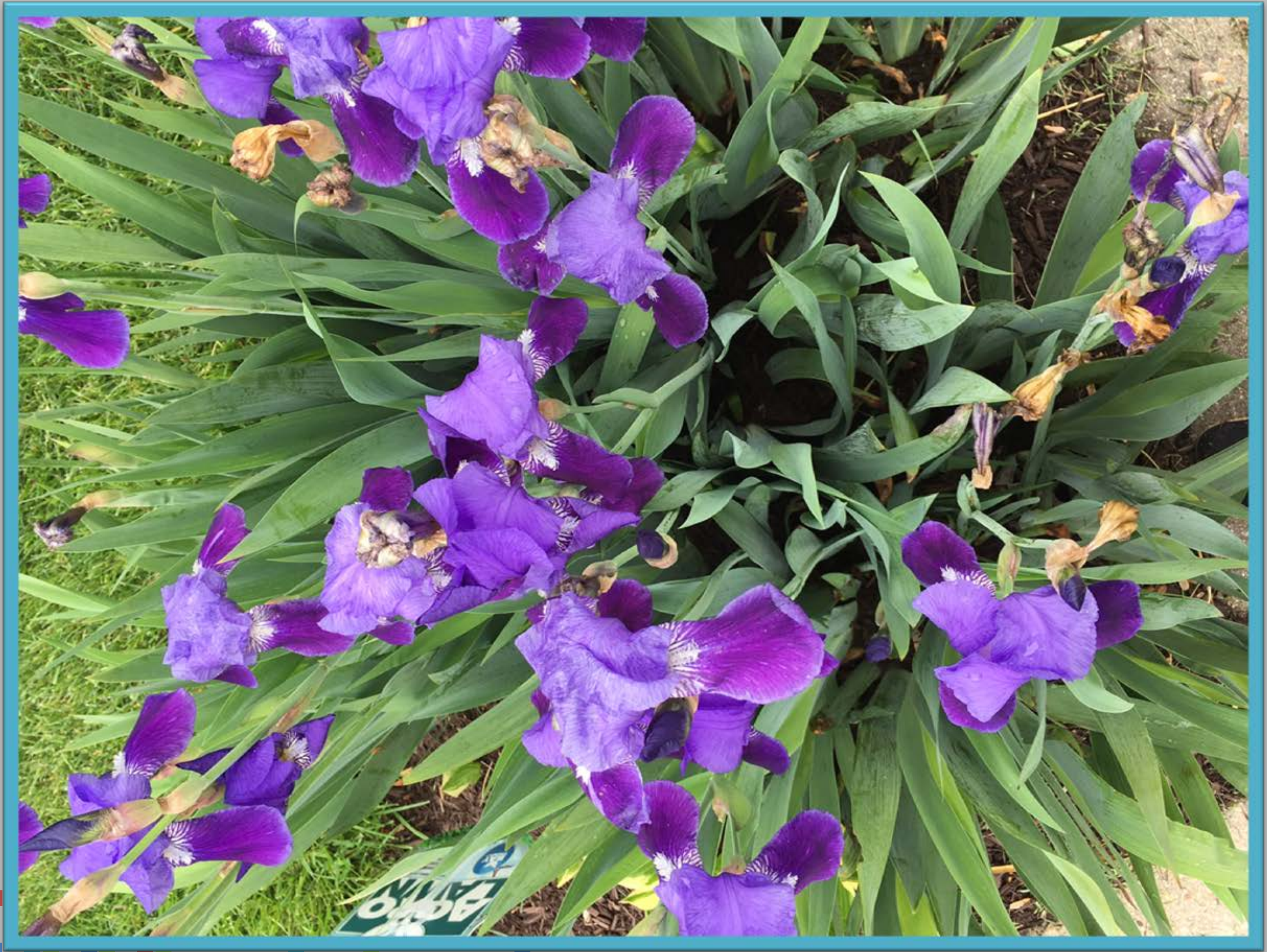

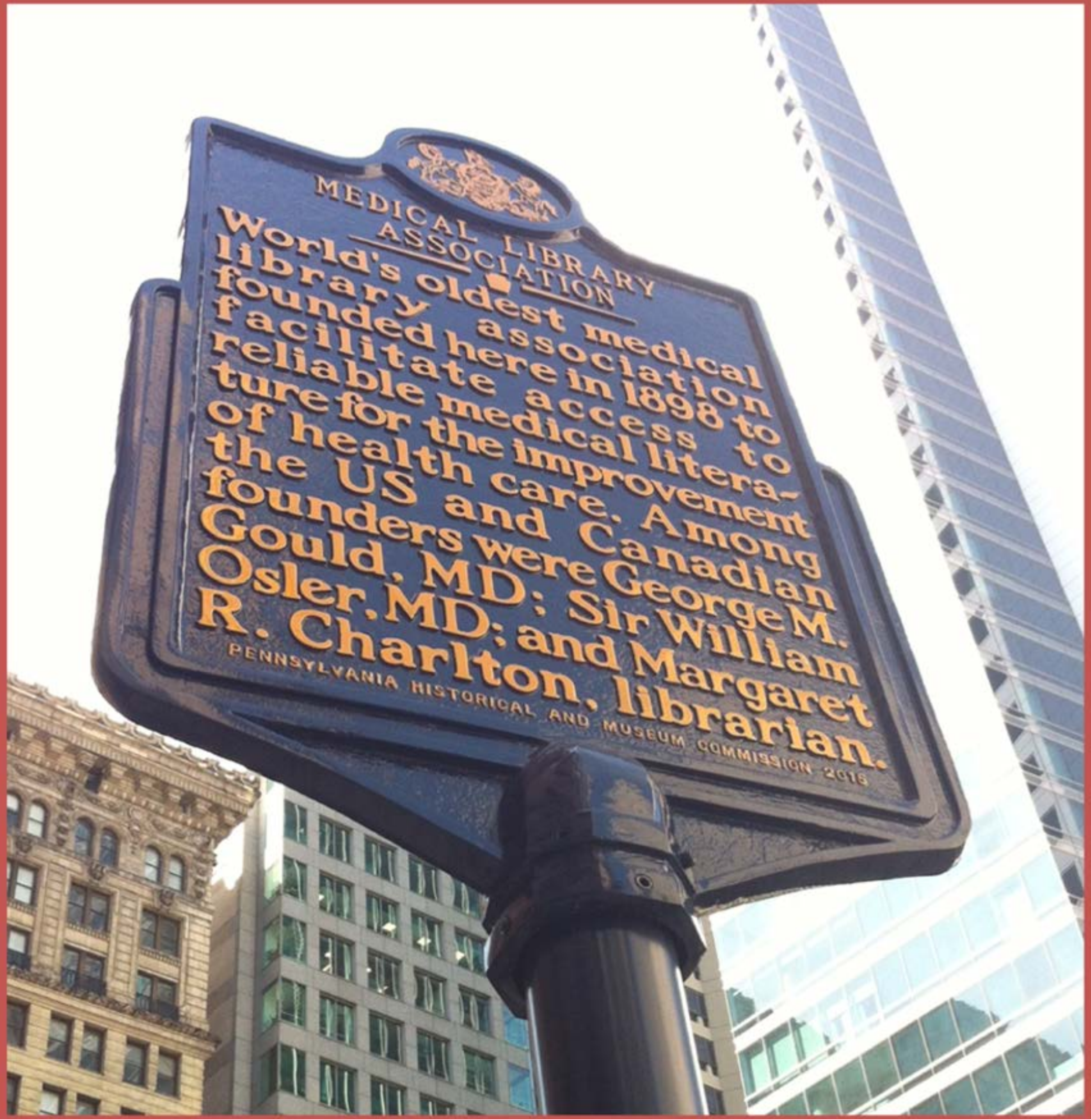
MEDICAL LIBRARY ASSOCIATION
World's oldest medical library association founded here in 1898 to facilitate access to reliable medical literature for the improvement of health care. Among the US and Canadian founders were George M. Gould, MD; Sir William Osler, MD; and Margaret R. Charlton, librarian.
PENNSYLVANIA HISTORICAL AND MUSEUM COMMISSION 2016

Marcia C. Noyes

1896 – Around the time she was hired at the Medical and Chirurgical Faculty of Maryland

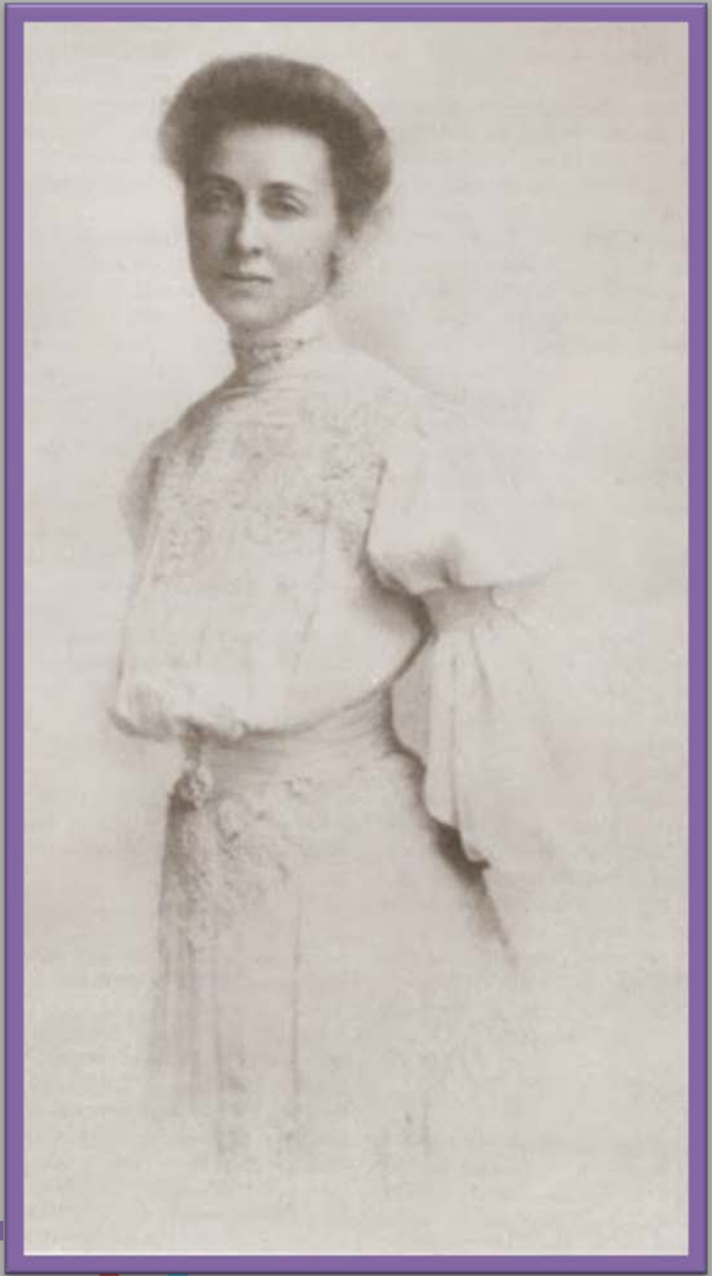

Marcia Noyes at the mid-point of her career and in her apartment at Med-Chi

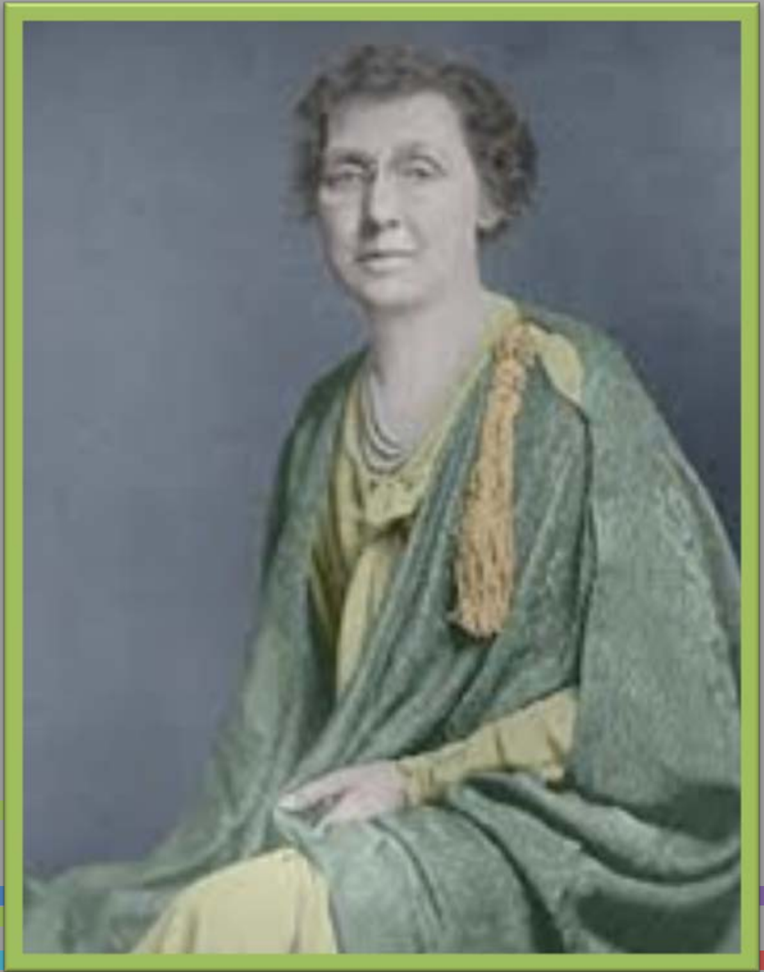

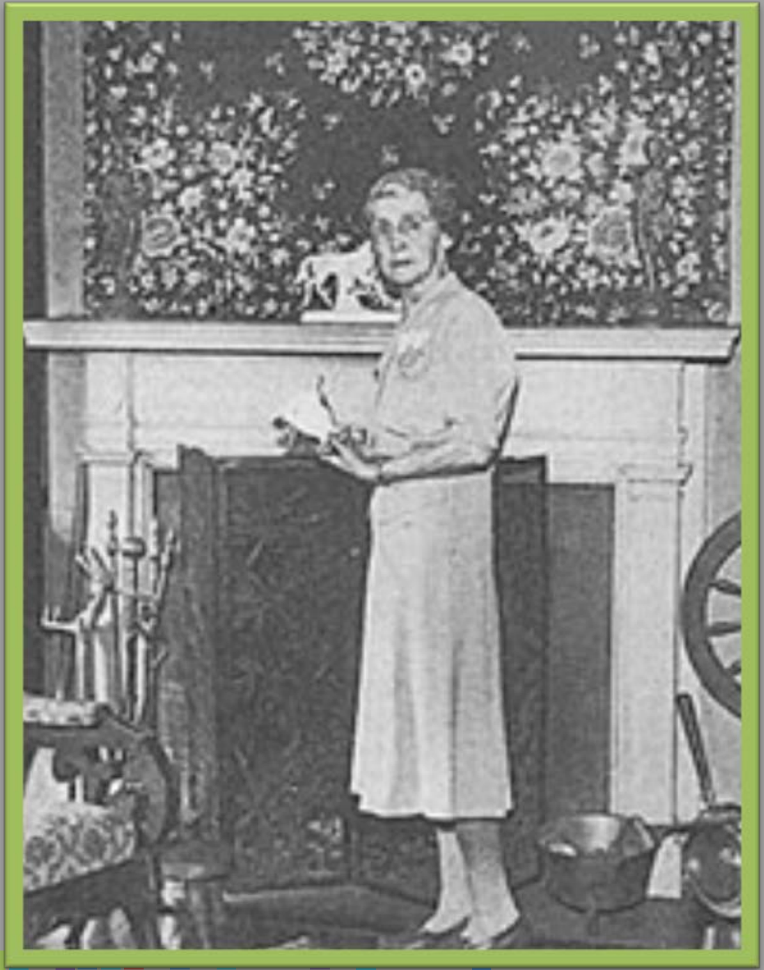

Green Mount Cemetery

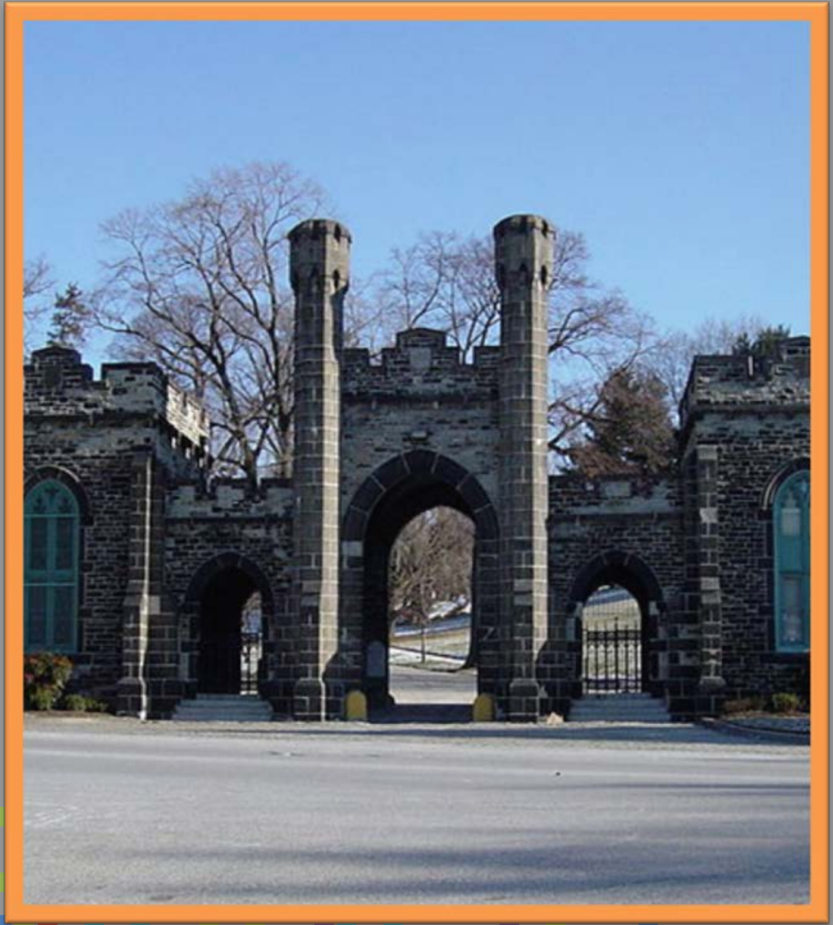

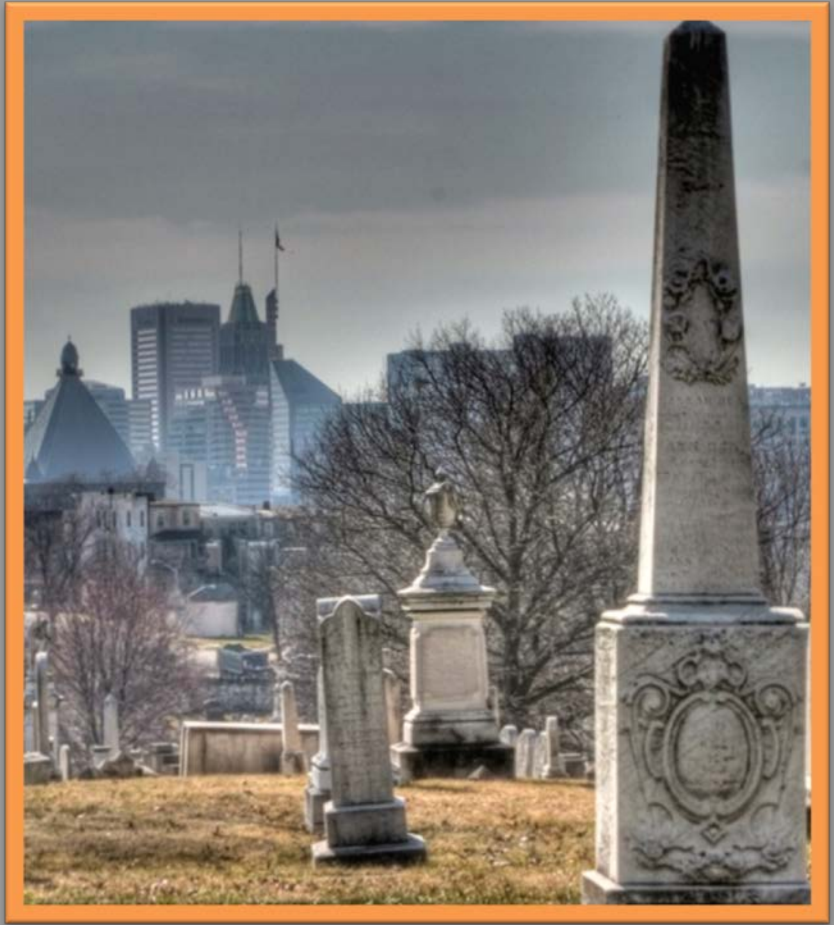

Making it right with Miss Noyes

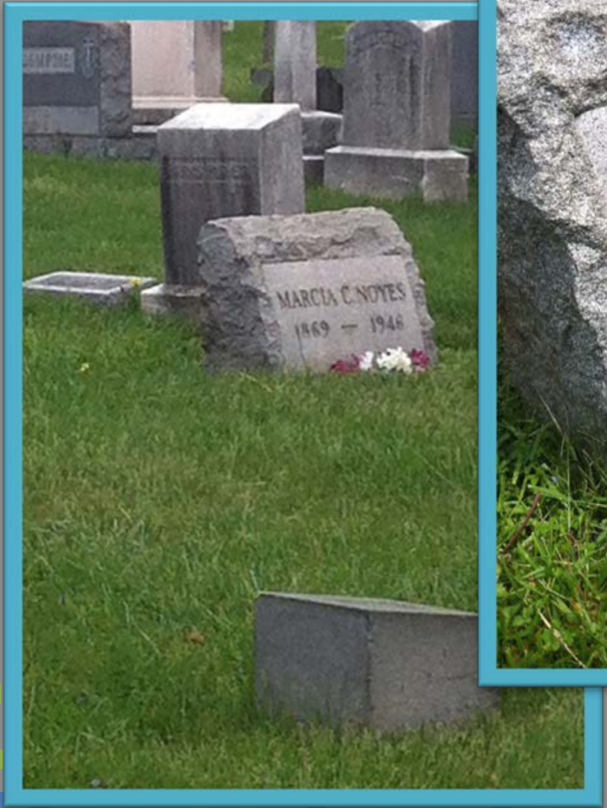

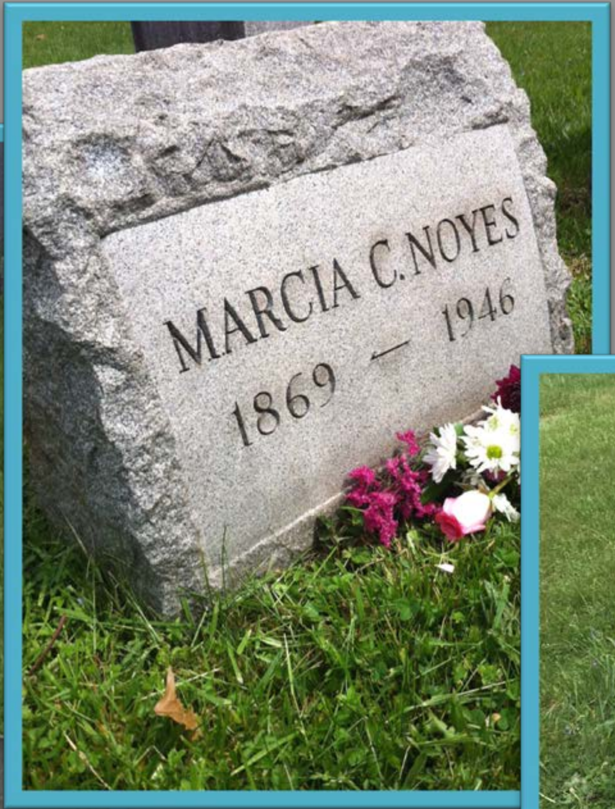

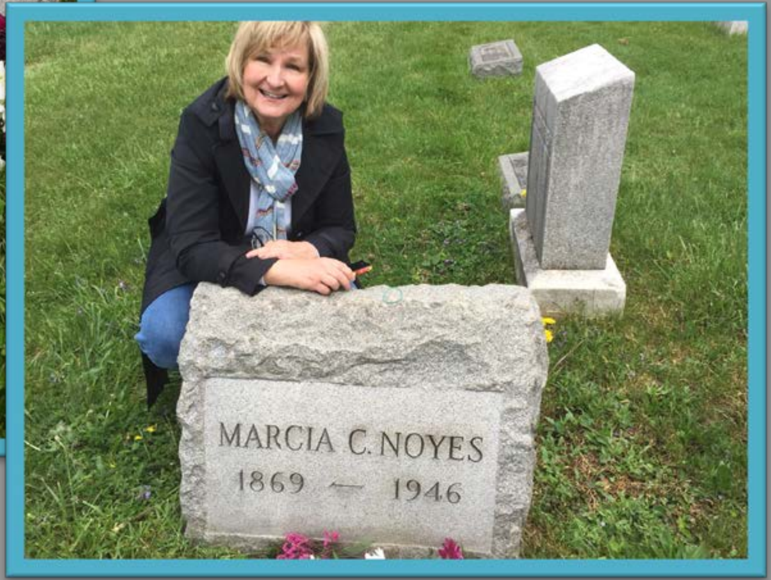

Leadership Characteristics – ALA New Members Roundtable

- Physical – energy, stamina
- Emotional – self aware, risk taker, concerned for others, persistent
- Social - motivator, collaborator/partner, listener
- Intellectual/Intelligence – adaptable, embraces ambiguity, lifelong learner
- Communication – articulate, one to one, one to many
- Experience – competent, participatory
- Trustworthy – caring, constant

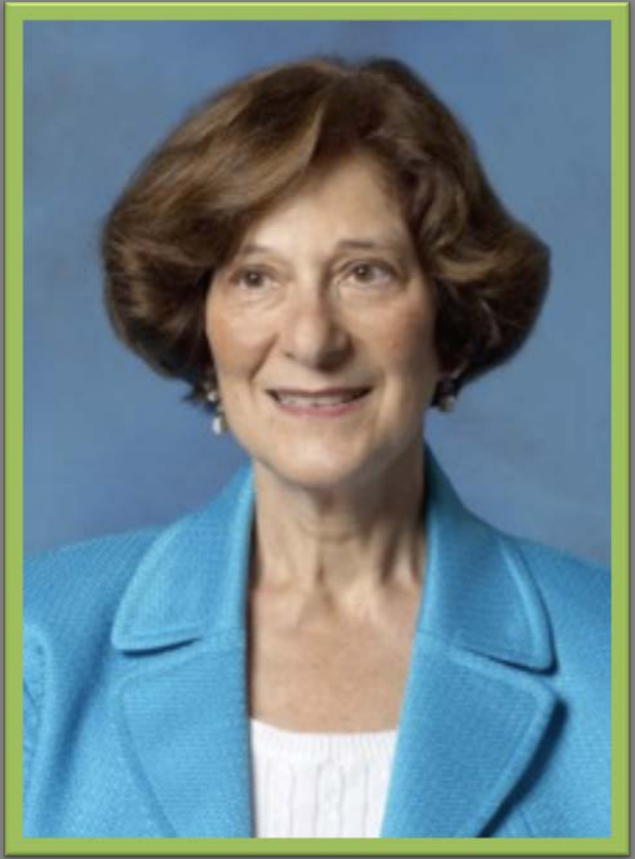

Barbara Epstein – 2015 Janet Doe Lecturer

"In Their Own Words: Oral Histories of Medical Library Association Past Presidents"

Text Analysis of MLA Presidential Profiles

58 profiles

64,373 words | 7,640 unique words

176 unique common words
accounted for 37,000 usages, removed

7,464 individual words remained

3,888 of those were only used once

Grouping

- Playing nice with others – cooperation, collegiality, collaboration – 10 terms combined for 45 occurrences
- Management – practical, effective, efficient – 22 terms combined for 56 occurrences
- Innovation – vision, agility, imagination – 20 terms combined for 75 occurrences
- Personal qualities – humor, stamina, passion – 51 terms combined for 212 occurrences
- And others: mentoring (44), ethics (20), politics (18)

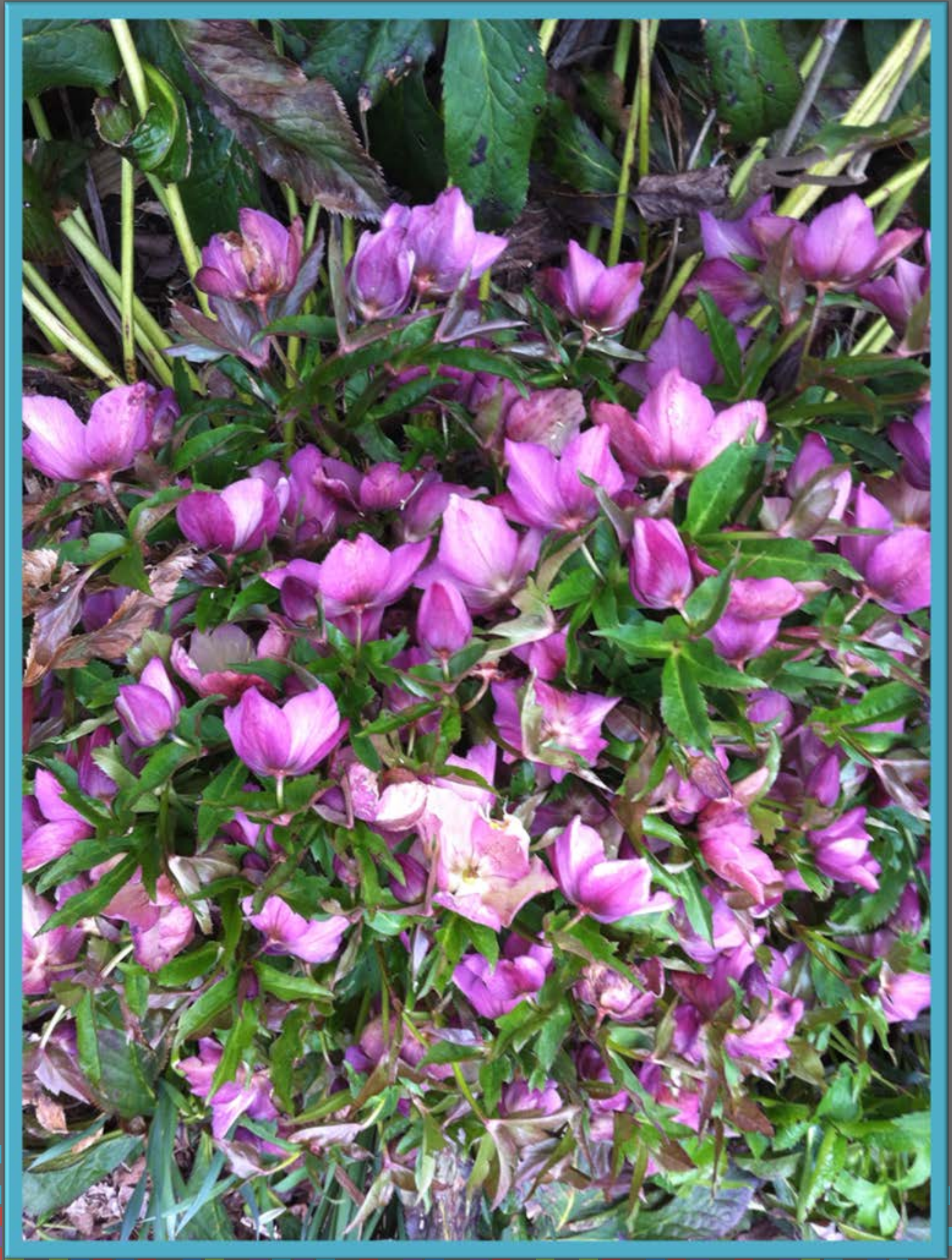

Questions – Past 25 Years of MLA Presidents
(78% response)

1. What was your path to the MLA presidency?
2. What leadership qualities did you bring to the presidency?
3. While president, what was your greatest leadership challenge?
4. What was the greatest lesson learned?
5. Have the leadership qualities needed for the MLA presidency changed?
6. What is the single most important trait for a leader to have?
7. Anything else you would like to share?

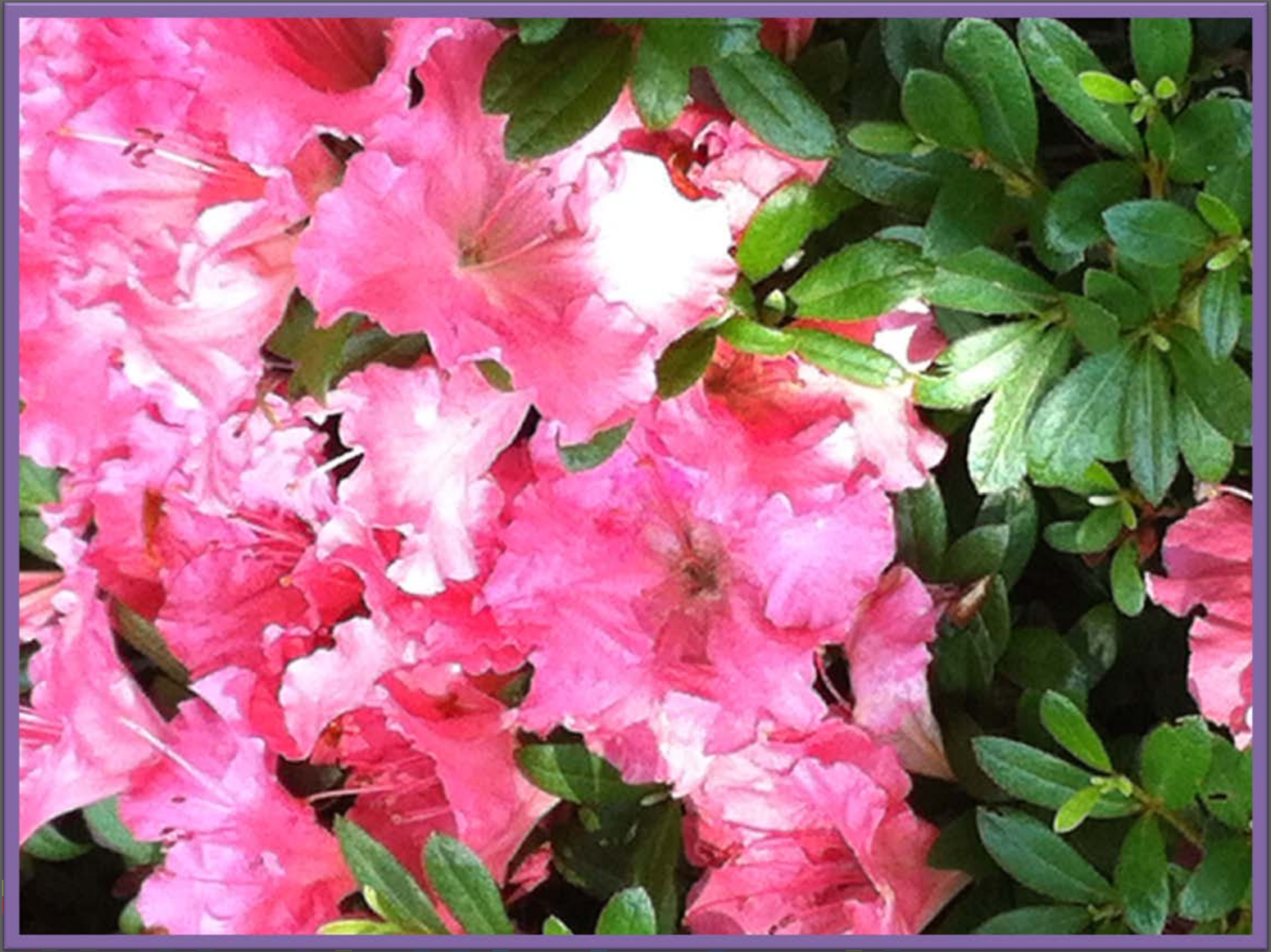

The Presidents

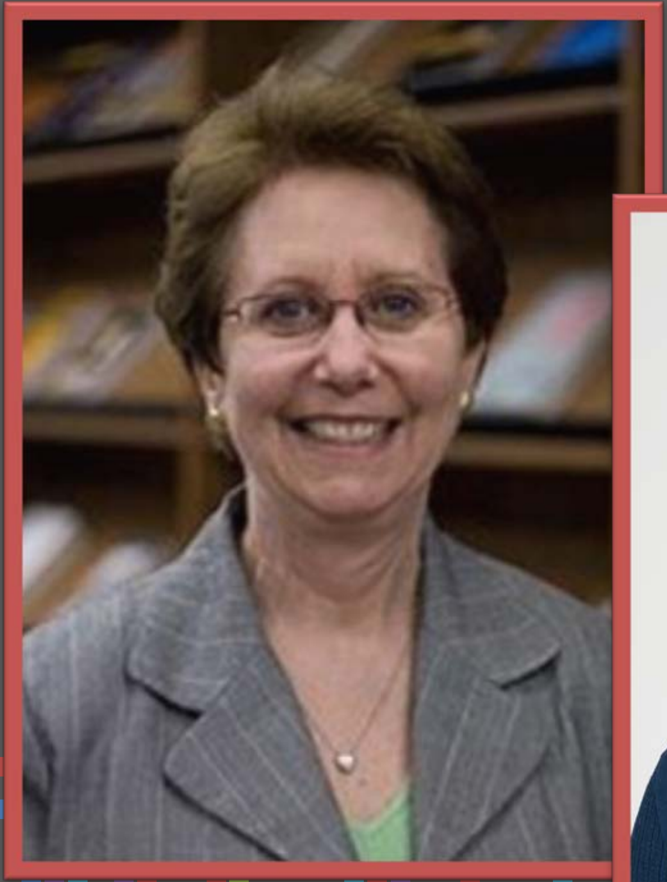

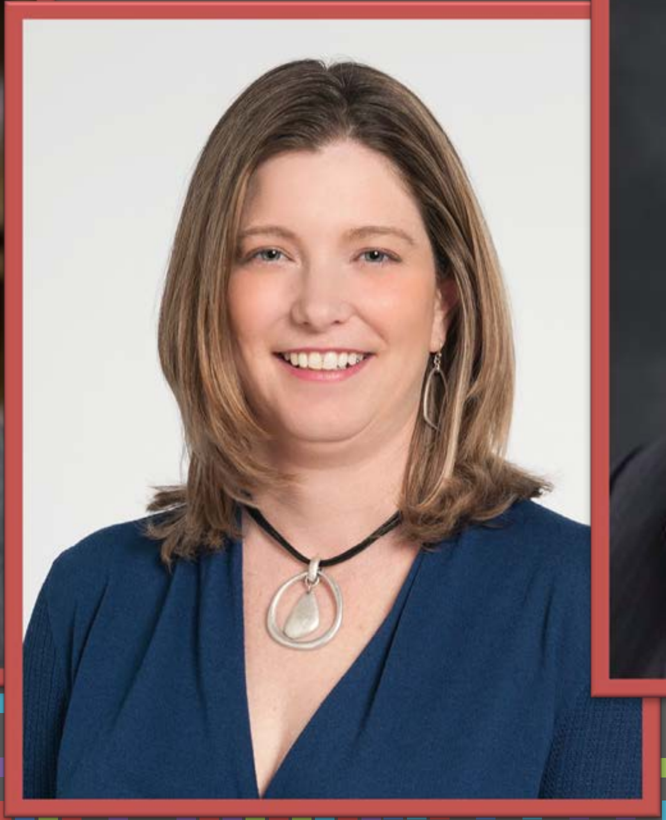

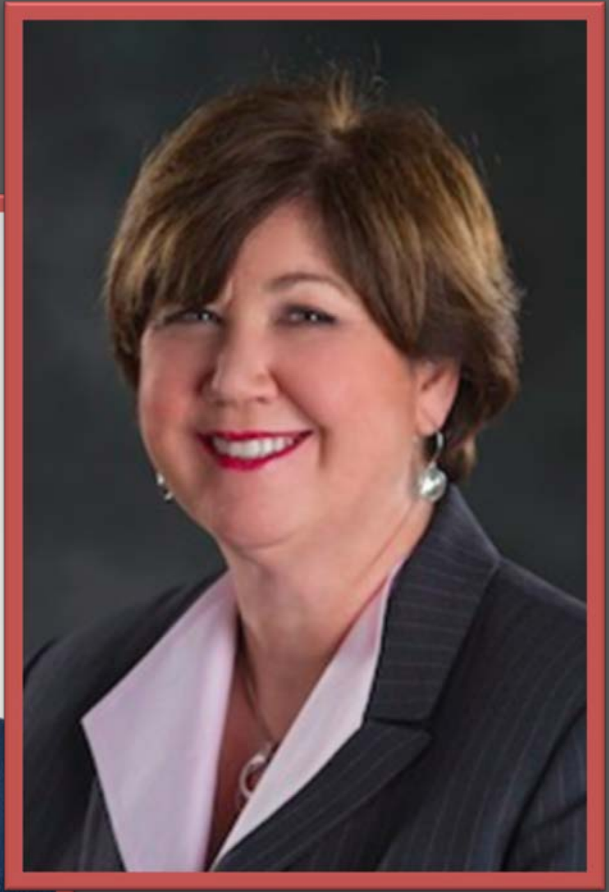

The Board

1997

- Visionary
- Communicator
- Knowledge of MLA/environment/issues
- Motivate, persuade, inspire
- Collaborator
- Energy, stamina, enthusiasm
- Respectful
- Open
- Politically aware
- Humble
- Comfort in the spotlight
- Passion for the profession

2016

- Communicator
- Visionary
- Interpersonal skills
- Self-confident
- Enthusiasm, energy, stamina
- Calm
- Trustworthy
- Respectful
- Humble
- Good sense of humor
- Problem solving skills
- Past leadership experience
- Passion for the profession

The Executive Directors

- Cheerleaders
- Facilitators
- Not ego-driven
- Humble
- Encouraging of different viewpoints
- Engagement of the members – inclusive
- Big picture view but saw the work to be done.

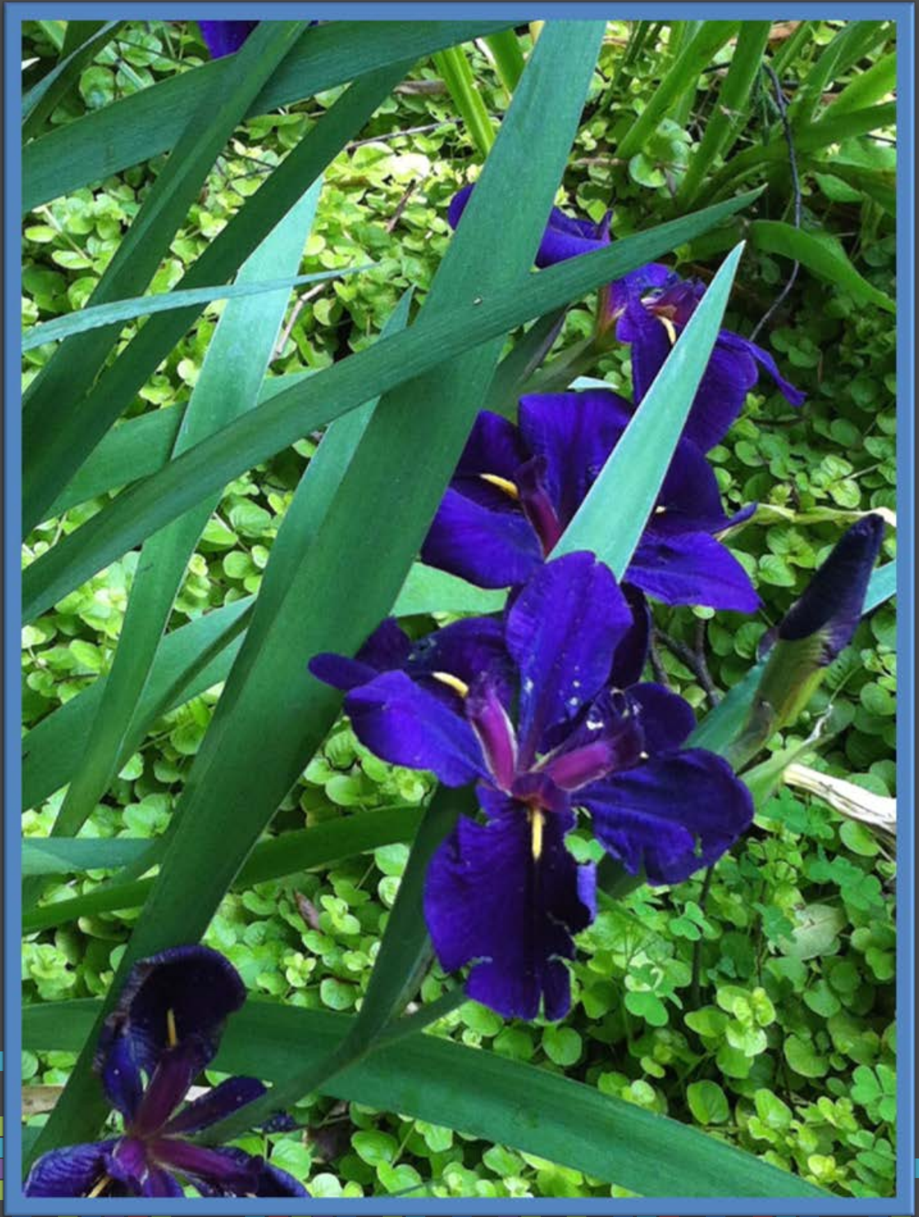

Future Leadership Traits

- Risk taking
- Innovative mind set
- Embrace, accept, and cause disruption
- Failure tolerant
- Resilience
- Empathy not sympathy
- Strong support networks

Lighting the spark…inhabiting the leadership role…MLA

Education

Awards and recognition

Experiential leader development

Mentoring

Kouzes and Posner – *Five Practices of Exemplary Leadership*

- Model the way
- Inspire a shared vision
- Challenge the process
- Enable others to act
- Encourage the heart

Leadership with a small “l”

Drew Dudley
2010 TEDx Toronto
“Everyday Leadership”

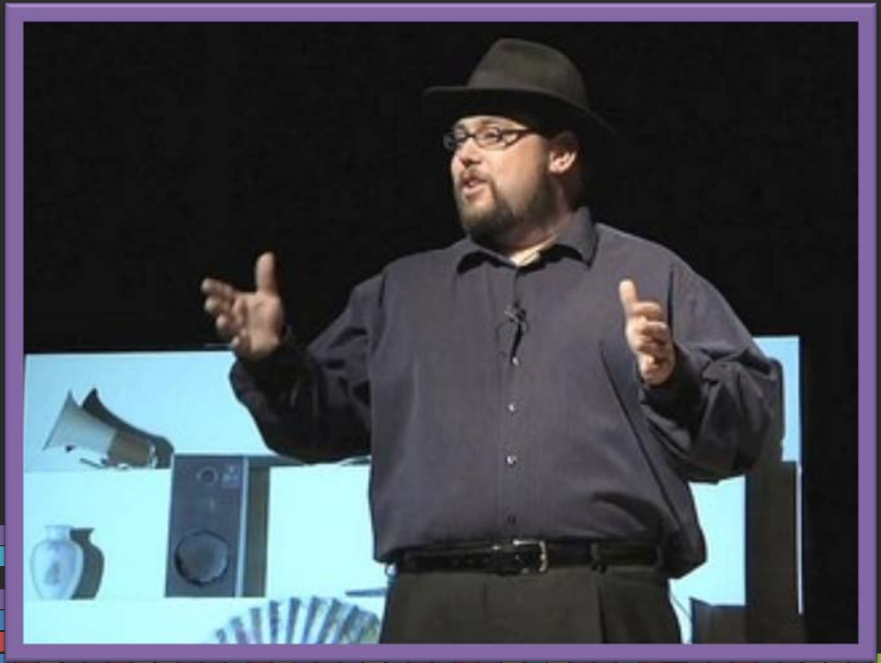

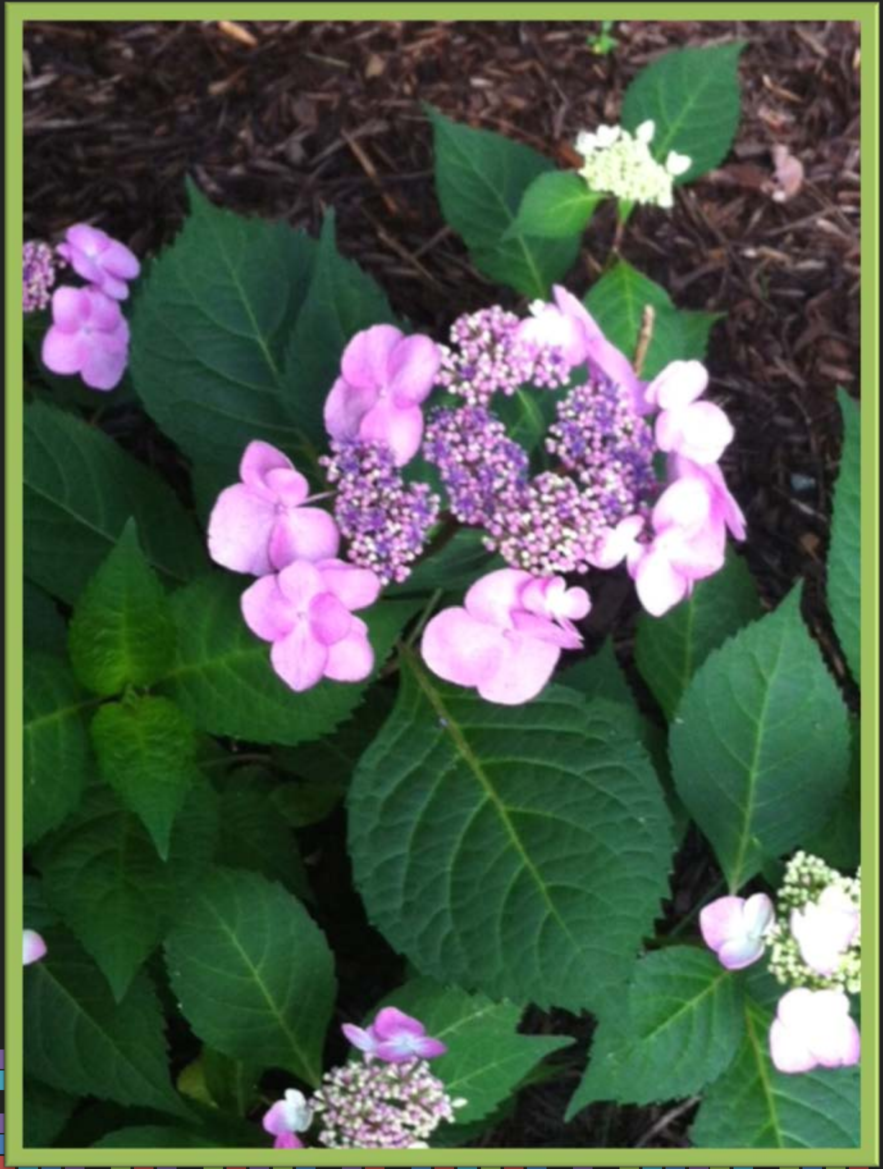

Thank you

We Can Be Heroes: MLA's Leadership Journey(s)
